# Supplementary material for: Marine and terrestrial contributions to atmospheric deposition fluxes of methylated arsenic species
Source: Nat Commun. 2024 Nov 7;15:9623. doi: 10.1038/s41467-024-53974-z (PMC11543862; doi:10.1038/s41467-024-53974-z)
Supplement: Supplementary file 1 — Supplementary Information [file 41467_2024_53974_MOESM1_ESM.pdf]

Supplementary Information

Marine and terrestrial contributions to atmospheric deposition fluxes of methylated arsenic species

Esther S. Breuninger[1,2*], Julie Tolu[1,2*], Franziska Aemisegger[3,4,5], Iris Thurnherr[3], Sylvain Bouchet[1,2], Adrien Mestrot[4,5], Rachele Ossola[2,6], Kristopher McNeill[2], Dariya Tukhmetova[7], Jochen Vogl[7], Björn Meermann[7], Jeroen E. Sonke[8], and Lenny H.E. Winkel[1,2*]

[1]Eawag, Swiss Federal Institute of Aquatic Science and Technology, Ueberlandstrasse 133, 8600 Dübendorf, Switzerland
[2]Institute of Biogeochemistry & Pollutant Dynamics, ETH Zurich, 8092 Zurich, Switzerland
[3]Institute for Atmospheric and Climate Science, ETH Zurich, 8092 Zurich, Switzerland
[4]Institute of Geography, University of Bern, 3012 Bern, Switzerland
[5]Oeschger Centre for Climate Change Research, University of Bern, 3012 Bern, Switzerland
[6]Department of Chemistry, Colorado State University, Fort Collins 80523, Colorado, United States
[7]Federal Institute for Materials Research and Testing, Division 1.1 - Inorganic Trace Analysis, 12489 Berlin, Germany
[8]Géosciences Environnement Toulouse, CNRS/IRD/Université de Toulouse, 31400 Toulouse, France

***correspondence to:** esther.breuninger@usys.ethz.ch; julie.tolu@eawag.ch; lenny.winkel@eawag.ch

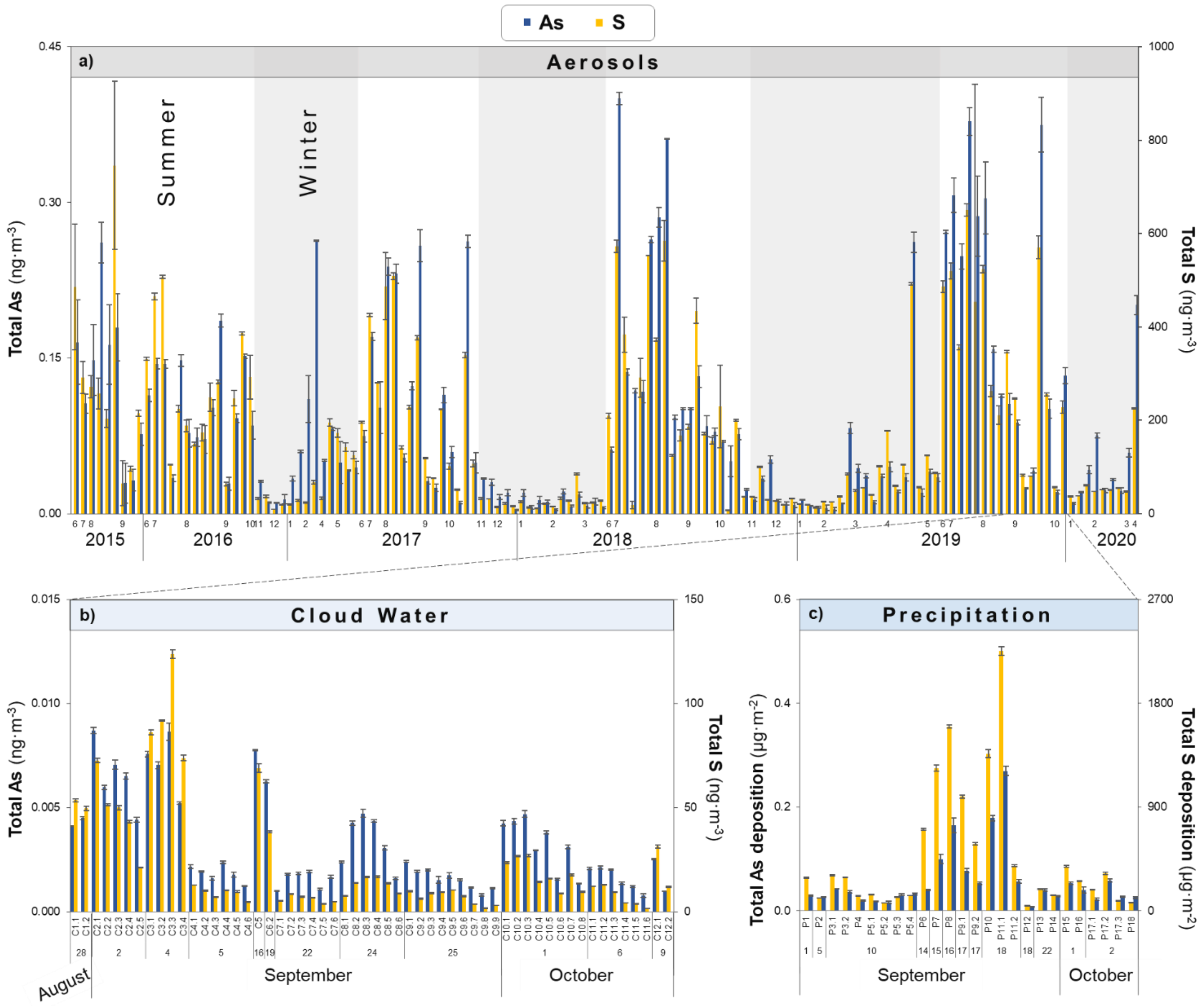

Supplementary Figure 1. Concentrations of total arsenic (As) and sulfur (S) over time for atmospheric samples collected in this study. Aerosol samples were collected at a weekly time resolution during the 2015-2020 (**a**, n=134). Cloud water (**b**, n=56) and precipitation (**c**, n=26) samples were collected at a sub-event basis during the 2019 campaign. The error bars in a-c represent the standard deviation values resulting from quantification by ICP-MS/MS in triplicate.

Supplementary Table 1. Overview of As species concentrations and deposition of sub-precipitation events. Indicated standard deviations represent the uncertainty of quantification by HPLC-ICP-MS/MS in duplicate.

	As species concentrations				As species depositions			
**Precipitation Sub-events**	**$TMAs^VO$** ($ng \cdot L^{-1}$)	**$DMAs^V$** ($ng \cdot L^{-1}$)	**$MMAs^V$** ($ng \cdot L^{-1}$)	**iAs** ($ng \cdot L^{-1}$)	**$TMAs^VO$** ($ng \cdot m^{-2}$)	**$DMAs^V$** ($ng \cdot m^{-2}$)	**$MMAs^V$** ($ng \cdot m^{-2}$)	**iAs** ($ng \cdot m^{-2}$)
P1	23.8 ± 0.3	3.1 ± 0.2	<LOD	52.4 ± 0.4	9.5 ± 0.1	1.2 ± 0.06	<LOD	20.9 ± 0.2
P2	18.7 ± 0.05	2.6 ± 0.3	<LOD	28.5 ± 0.06	10.4 ± 0.02	1.4 ± 0.1	<LOD	15.8 ± 0.03
P3.1	2.7 ± 0.08	0.9 ± 0.03	<LOD	14.4 ± 0.04	5.8 ± 0.2	1.8 ± 0.06	<LOD	30.2 ± 0.09
P3.2	1.9 ± 0.1	0.9 ± 0.1	<LOD	12.1 ± 0.2	4.0 ± 0.2	1.9 ± 0.2	<LOD	25.3 ± 0.4
P4	1.9 ± 0.2	1.0 ± 0.06	<LOD	19.2 ± 0.7	1.8 ± 0.2	0.9 ± 0.05	<LOD	18.1 ± 0.7
P5.1	2.7 ± 0.04	1.3 ± 0.1	<LOD	5.8 ± 0.02	4.3 ± 0.07	2.0 ± 0.2	<LOD	9.2 ± 0.03
P5.2	1.6 ± 0.2	0.9 ± 0.1	<LOD	6.0 ± 0.1	2.2 ± 0.2	1.3 ± 0.2	<LOD	8.1 ± 0.2
P5.3	2.4 ± 0.1	0.9 ± 0.04	<LOD	11.6 ± 0.03	4.2 ± 0.2	1.6 ± 0.07	<LOD	20.2 ± 0.05
P5.4	3.6 ± 0.09	1.1 ± 0.09	<LOD	16.8 ± 0.4	4.4 ± 0.1	1.3 ± 0.1	<LOD	20.7 ± 0.5
P6	6.3 ± 0.1	1.8 ± 0.1	0.6 ± 0.8	40.1 ± 0.4	4.6 ± 0.1	1.3 ± 0.08	0.4 ± 0.6	29.3 ± 0.3
P7	2.8 ± 0.1	1.0 ± 0.1	<LOD	23.2 ± 0.5	10.0 ± 0.3	3.5 ± 0.5	<LOD	82.7 ± 1.7
P8	11.2 ± 0.05	2.5 ± 0.1	<LOD	31.3 ± 0.08	42.2 ± 0.2	9.4 ± 0.4	<LOD	117.8 ± 0.3
P9.1	5.2 ± 0.8	1.8 ± 0.1	0.1 ± 0.2	27.9 ± 0.3	7.1 ± 1.1	2.5 ± 0.1	0.2 ± 0.2	38.4 ± 0.5
P9.2	6.3 ± 0.2	1.4 ± 0.09	0.6 ± 0.8	35.6 ± 0.6	6.2 ± 0.2	1.4 ± 0.09	0.6 ± 0.8	35.2 ± 0.6
P10	7.7 ± 0.4	1.2 ± 0.02	<LOD	24.5 ± 0.1	34.0 ± 1.9	5.5 ± 0.1	<LOD	108.1 ± 0.6
P11.1	8.0 ± 0.05	1.9 ± 0.02	<LOD	29.8 ± 0.06	52.3 ± 0.3	12.6 ± 0.1	<LOD	193.5 ± 0.4
P11.2	3.6 ± 0.2	1.0 ± 0.07	<LOD	20.8 ± 0.3	8.5 ± 0.5	2.5 ± 0.2	<LOD	49.7 ± 0.6
P12	9.5 ± 0.2	2.5 ± 0.03	<LOD	17.0 ± 0.1	2.2 ± 0.03	0.6 ± 0.01	<LOD	3.9 ± 0.03
P13	17.9 ± 0.2	2.4 ± 0.1	<LOD	27.3 ± 0.4	15.7 ± 0.2	2.1 ± 0.1	<LOD	23.9 ± 0.3
P14	18.7 ± 0.3	3.1 ± 0.1	<LOD	29.4 ± 0.4	10.9 ± 0.2	1.8 ± 0.06	<LOD	17.2 ± 0.3
P15	6.3 ± 0.1	1.3 ± 0.03	<LOD	15.5 ± 0.2	13.3 ± 0.2	2.8 ± 0.06	<LOD	32.7 ± 0.5
P16	6.2 ± 0.2	1.4 ± 0.03	<LOD	11.2 ± 0.6	12.7 ± 0.4	2.9 ± 0.06	<LOD	22.9 ± 1.2
P17.1	3.2 ± 0.05	1.1 ± 0.09	<LOD	6.8 ± 0.3	6.4 ± 0.1	2.2 ± 0.2	<LOD	13.7 ± 0.5
P17.2	2.4 ± 0.01	0.9 ± 0.1	<LOD	6.2 ± 0.4	13.7 ± 0.04	5.2 ± 0.9	<LOD	35.5 ± 2.1
P17.3	1.8 ± 0.1	0.7 ± 0.02	<LOD	12.9 ± 0.2	2.8 ± 0.2	1.1 ± 0.04	<LOD	20.3 ± 0.4
P18	3.4 ± 0.1	1.1 ± 0.1	<LOD	13.0 ± 0.4	4.4 ± 0.1	1.5 ± 0.1	<LOD	17.0 ± 0.5

Supplementary Discussion 1: Extraction and pre-concentration of As species in atmospheric deposition

a) Pre-concentration of As species by lyophilization

Aqueous atmospheric samples, including precipitation and cloud water samples and aerosol water extracts (detailed description in the next section), were pre-concentrated using the lyophilization method previously developed for selenium (Se)[1].

Briefly, the pre-concentration protocol included the lyophilization from an initial volume of 12 mL to a residual volume of 1.5 mL (pre-concentration factor of 8), to which 2 mmol·L^{-1} ammonium citrate was added to increase ionic strength. The suitability of the method in terms of As species recovery and stability was tested on three different rainwater samples collected at Pic du Midi Observatory (previously stored at 4°C) and spiked with individual and mixtures of methylated (i.e., MMAs^{V}, DMAs^{V}) and inorganic As species (i.e., As^{V}) previously identified in rainwater[2, 3, 4] and aerosols[5, 6, 7, 8]. Recoveries were determined by comparing the initial concentration of spiked As species (200 ng·L^{-1}) to the final concentrations after lyophilization. Analyses were performed via HPLC-ICP-MS/MS (described in Supplementary Discussion 2).

Supplementary Figure 2 demonstrates that all As species tested were fully recovered after lyophilisation (MMAs^{V}: 103 ± 2%, DMAs^{V}: 96 ± 4% As^{V}: 103 ± 4%), regardless of whether a compound was spiked individually or as a species mixture.

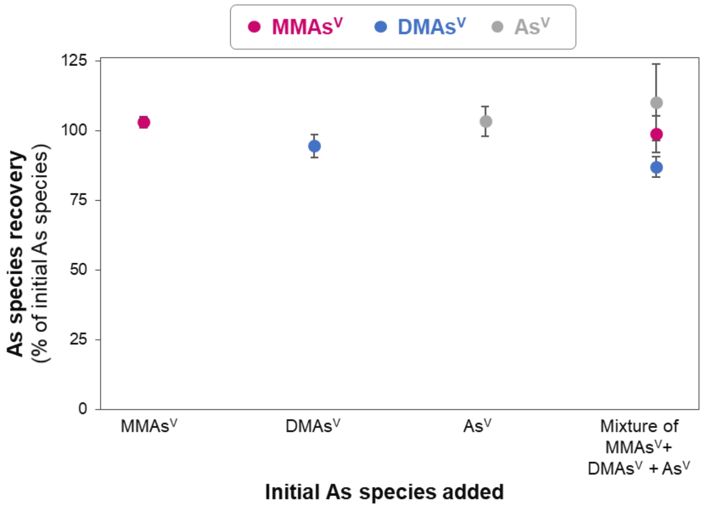

Supplementary Figure 2. As species recoveries during lyophilisation in rainwaters collected at Pic du Midi Observatory. Recoveries of initial added single As species (MMAs^{V}, DMAs^{V}, As^{V}) and As species mixture (MMAs^{V}+DMAs^{V}+As^{V}) are given in % of initial As species concentrations added (200 ng·L^{-1}). The error bars represent the standard deviation values of three independent rainwaters injected each two times, so a total of 6 analytical replicates.

b) Aerosol extraction

Two different extraction methods were applied to collected aerosol samples to determine (i) As species in the water soluble fraction by extraction in ultrapure water and (ii) total As species by extraction in 1% HNO_3 (previously applied to rice and other biological samples)[9].

Similar to a method previously described for Se[1], As species extraction in the water soluble fraction involved placing 11.404 cm^2 of aerosol filter (corresponding to 7197 ± 1816 m^3 of sampled air) in a polypropylene tube with 15 mL of ultrapure water. The mixture was then sonicated twice for 20 min at 20°C. Sonication was performed twice for 20 min (instead of once for 40 min) to avoid increasing the sample's temperature. After sonication, the supernatant

was filtered (0.22 µm syringe filter, Nylon 66, BGB) and stored at -20°C until pre-concentration by lyophilization (described in previous section).

The stability of $MMAs^V$, $DMAs^V$, $TMAs^VO$, and As^V during this process was determined by measuring recoveries of spike compounds (0.5-10 $µg·L^{-1}$) following sonication by HPLC-ICP-MS/MS (described in Supplementary Discussion 2). $MMAs^V$, $DMAs^V$, $TMAs^VO$, and As^V showed high recoveries, i.e., 96 ± 6%, 97 ± 1%, 98 ± 3% and 97 ± 1%, respectively.

To quantify total As species, the extraction protocol included the addition of 4.8 mL 1% (*v/v*) of 69% HNO_3 and 0.2 mL H_2O_2 (30%, (*w/w*)) with an extraction ratio of 14.137 $cm^2·5\ mL^{-1}$. Samples were extracted in a microwave oven (UltraClave IV, MLS) according to Sun et al.[10]. The extraction program included a first temperature increase to 55°C (5 min ramp, temperature held for 10 min) then to 75°C (5 min ramp, temperature held for 10 min) and finally to 95°C (5 min ramp, temperature held for 30 min). After extraction, the suspensions were filtered (0.22 µm syringe filter, Nylon 66, BGB) and stored at 4°C until analysis.

We determined the extraction efficiencies of the two methods by comparing the sum of detected species to the concentration of total As in acid digestions obtained by ICP-MS/MS analysis (using HNO_3 and H_2O_2). Extraction efficiencies in water and 1% HNO_3 extracts were 38±17% (n=70) and 87±33% (n=70), respectively. For comparison, S species were fully recovered by water extraction (103±23%).

Apart from ultrapure water extractions, several previous authors used harsher extraction procedures, with average efficiencies ranging from 42 to 89% (using ultrapure water[11], diluted H_2O_2[5, 6, 7, 8] or hydroxylammonium chloride[12]) – although we note that these studies were performed on aerosol samples with ~3-7 times[5, 6, 7] or even up to ~50-70 times[11, 12] higher As concentration compared to ours. Extraction efficiencies may also vary depending on the particle size fraction containing As, which is likely to be source dependent. Indeed, previous studies reported higher recoveries of As species for smaller size fractions of urban aerosols[7, 11].

Supplementary Discussion 2: HPLC-ICP-MS/MS method optimization and detailed procedures

The chromatographic separation of As species was optimized on the basis of the method published by Raber et al.[13] that used anion exchange chromatography (Hamilton PRP-X100, 150×4.1 mm, 5 µm) with isocratic elution of malonic acid (10 $mmol·L^{-1}$, pH 5.6) delivered at 1 $mL·min^{-1}$, and injection volumes of 10-20 µL[13].

Here, we used the same column type, but with smaller inner column diameter (i.e., 2.1 mm versus 4.1 mm) to reduce solvent consumption (reduction of mobile phase flow rate). Furthermore, we optimized the isocratic elution of the tested As species by using a lower solvent concentration in combination with a flow gradient (see Supplementary Table 2). This choice allowed us to improve peak shapes, thereby decreasing the detection limits of As species. To further improve peak shapes, we used a Peltier Cooled Cyclonic Chamber (PC3-MR, Element Scientific) equipped with a PFA MicroFlow (Element Scientific).

Supplementary Table 2. Flow gradient of the HPLC-ICP-MS/MS method used for the speciation analysis of As.

**Time** (min)	**Malonic acid** (mmol·L^{-1})	**Flow** (mL·L^{-1})
1.6	5	0.4
1.7	5	0.5
3.5	5	0.5

Supplementary Figure 3 shows the HPLC-ICP-MS/MS chromatogram obtained with a mixed standard solution of 1 µg·L^{-1} using our optimized separation (Supplementary Figure 3b) and using the method of Raber et al.[13] (obtained with a mixed standard solution of 10 µg·L^{-1}, Supplementary Figure 3a). In comparison to the previous method by Raber et al.[13], species separation and peak shape was improved and consumption of solvent (0.5 mL·min^{-1}) was significantly reduced.

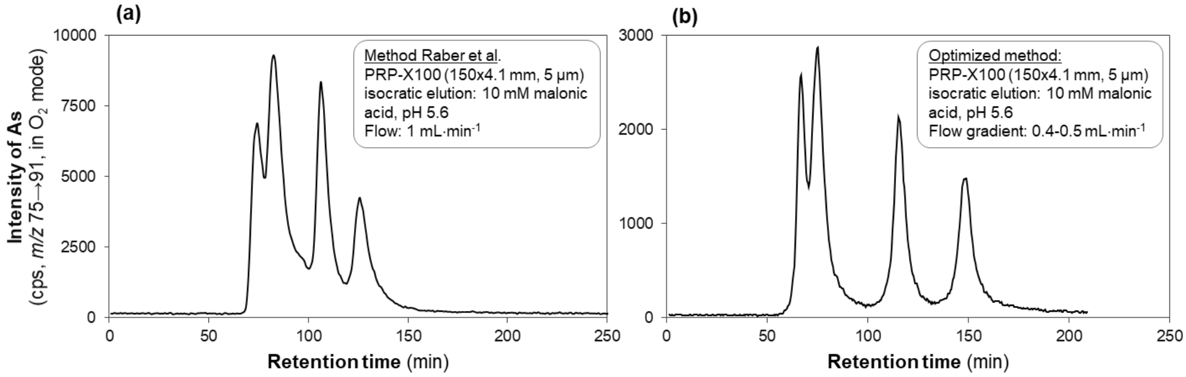

Supplementary Figure 3. Comparison of As intensity chromatograms obtained for mixed standard solutions for initial HPLC-ICP-MS method described by Raber et al.[13] (a) and with the optimized HPLC-ICP-MS/MS method (b). The mixed As species standard solutions contained 10 µg(As)·L^{-1} (a, method Raber et al.) or 1 µg(As)·L^{-1} (b, optimized method) of TMAs^{V}O, DMAs^{V}, MMAs^{V}, and As^{V} (in order of elution). The initial HPLC-ICP-MS method described by Raber et al.[13] (a) involves a PRP-X100 column (150x4.1 mm, 10 µm), an isocratic elution using 10 mmol·L^{-1} malonic acid at pH 5.6, a flow of 1 mL·min^{-1} and an injection volume of 10 µL. Our optimized HPLC-ICP-MS/MS method (b) involves a PRP-X100 column (150x2.1 mm, 5 µm), isocratic elution using 5 mmol·L^{-1} malonic acid at pH 5.6, a flow gradient of 0.4-0.5 mL·min^{-1} and an injection volume of 20 µL. Shown chromatograms were obtained without adding tetramethylammonium hydroxide (TMAH) post-column.

To further increase the sensitivity of As analyses via the well-known carbon enhancement effect[14], 14% (*v/v*) tetramethylammonium hydroxide (TMAH) was continuously added post-column to the internal standard solution through a T-piece via the peristaltic pump of the ICP-MS/MS. The addition of TMAH resulted in an increase of sensitivity of approximately 3.5. With this set-up and considering the pre-concentration step, detection limits of 1-2 ng·L^{-1} were reached, depending on the As species.

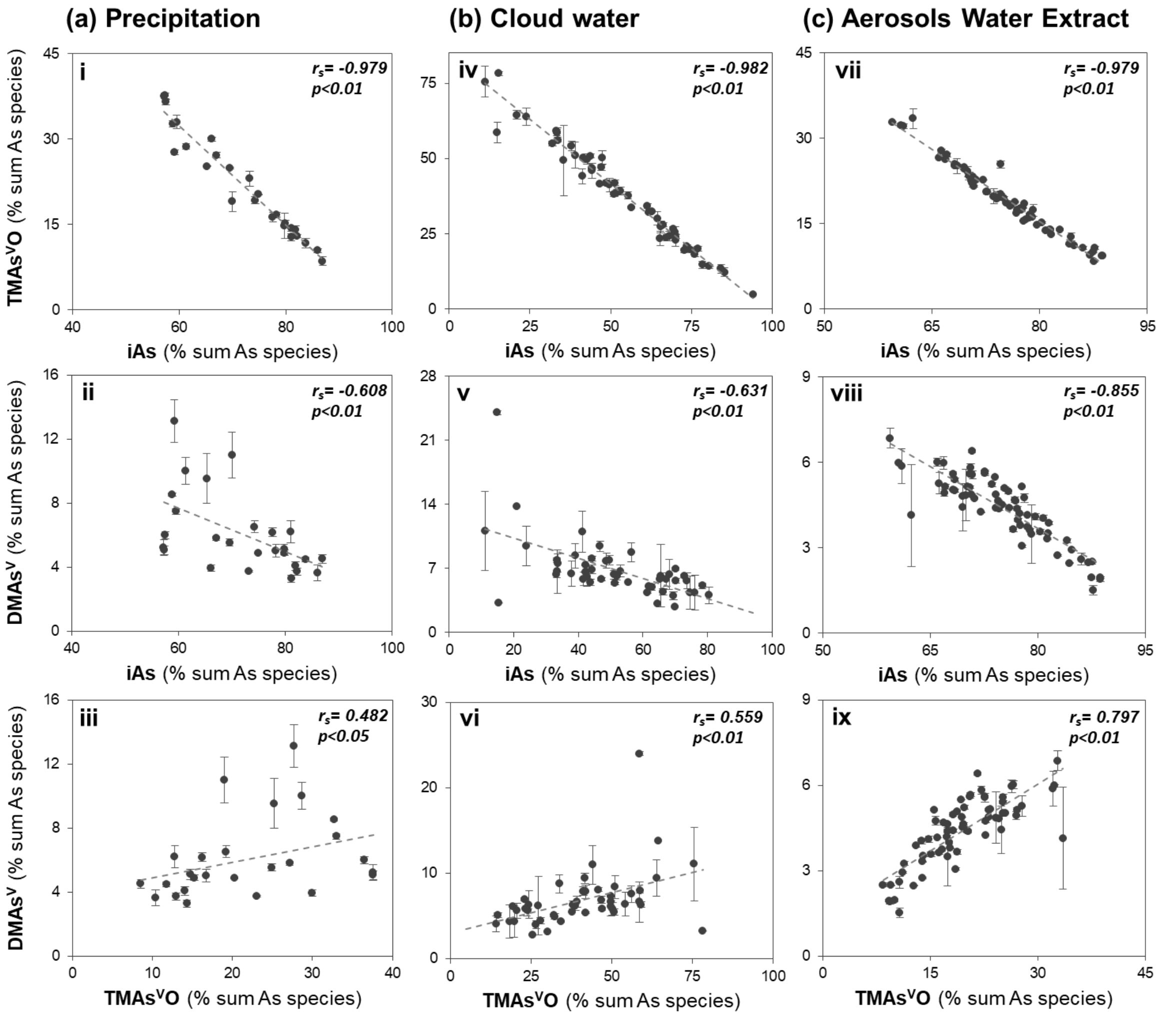

Supplementary Figure 4. **Bi-variate correlations between As species proportions in precipitation (panel a), cloud water (panel b) and aerosol water extracts (panel c).** The top and middle panels show the correlations between the proportions of inorganic As and $TMAs^VO$ (panel i: precipitation, panel iv: cloud water, and panel vii: aerosol extract) or between inorganic As and $DMAs^V$ (panel ii: precipitation, panel v: cloud water, and panel viii: aerosol extract). The bottom panels show the correlations between the proportions of $DMAs^V$ and $TMAs^VO$ (panel iii: precipitation, panel vi: cloud water, and panel ix: aerosol extract). Indicated correlation coefficients for precipitation (n= 26, $p<0.05$), cloud water (n= 55, $p<0.01$) and aerosol water extracts (n= 70, $p<0.01$) are significant, and the error bars represent the uncertainty of quantification by HPLC-ICP-MS/MS in duplicate, with invisible error bars indicating standard deviations within the symbol.

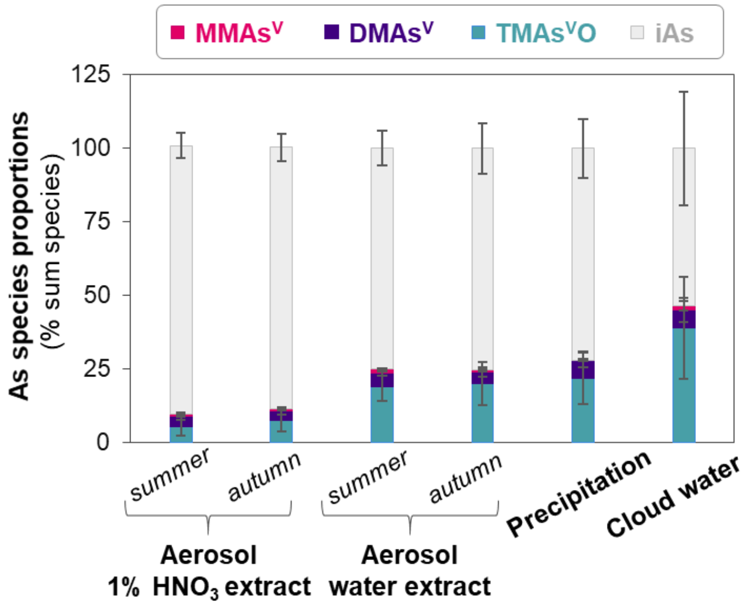

Supplementary Figure 5. Variability of proportions of As species in different atmospheric deposition samples. Species proportions (% sum As species concentrations in respective extracts or sample types) of MMAs^{V} (pink), DMAs^{V} (purple), TMAs^{V}O (cyan) and iAs (grey) in the 1% HNO_3 aerosol extract, the aerosol water extract, as well as in precipitation and cloud water. As species proportions in both aerosol extracts are divided into summer (June-August; 2015-2019) and autumn (September-October; 2015-2019) months. Precipitation and cloud water samples were collected in autumn months (campaign September-October; 2019).

Supplementary Table 3. Averages and standard deviations of moisture sources within 4 main groups of precipitation (sub)events identified by hierarchical cluster analysis. Identified cluster discussed in the main manuscript include: cluster 1: predominant moisture sources from Spain/ France, cluster 2: predominant moisture sources from North Atlantic, cluster 3: predominant moisture sources from North Africa/ Mediterranean Sea, cluster 4: predominant moisture sources from Atlantic/Spain. Considered sources regions are classified by land (sub-regions: UK, Ireland, Spain, Portugal, France, Local (local source, in France south of 43.6°N), Eastern Europe, Africa, America and other lands) and oceanic moisture sources (sub-regions: Atlantic, Bay of Biscay, Atlantic Subtropics, Western and Eastern Mediterranean Sea, Arctic and other seas). ∑ indicates the sum of moisture contributions of sub-regions over land and the ocean, respectively.

	Cluster 1	Cluster 2	Cluster 3	Cluster 4
**Land**	∑ 72.3 ± 8.7	∑ 36.0 ± 16.3	∑ 69.8 ± 3.5	∑ 45.0 ± 4.4
UK	0.2 ± 0.1	0.7 ± 0.7	0.1 ± 0.1	0.0 ± 0.0
Ireland	0.0 ± 0.0	1.4 ± 1.0	0.0 ± 0.0	0.0 ± 0.0
Spain	40.2 ± 5.4	9.2 ± 5.5	18.0 ± 1.9	26.9 ± 1.9
Portugal	2.5 ± 3.0	0.7 ± 0.9	0.1 ± 0.1	3.6 ± 1.7
France	16.2 ± 4.0	18.1 ± 13.1	15.6 ± 4.2	7.0 ± 2.1
Local	9.1 ± 2.7	2.6 ± 1.2	8.0 ± 0.7	3.3 ± 0.7
Eastern Europe	1.3 ± 1.1	0.0 ± 0.1	2.4 ± 0.9	0.5 ± 0.7
Africa	2.8 ± 1.7	0.0 ± 0.0	25.6 ± 8.5	0.7 ± 0.8
America	0.2 ± 0.4	3.1 ± 2.0	0.0 ± 0.0	3.0 ± 0.5
Other Lands	0.0 ± 0.0	0.1 ± 0.1	0.0 ± 0.0	0.0 ± 0.0
**Ocean**	∑ 26.9 ± 8.8	∑ 63.6 ± 16.4	∑ 28.4 ± 3.6	∑ 54.4 ± 4.2
Atlantic	10.9 ± 3.6	46.8 ± 18.1	3.6 ± 0.4	45.5 ± 4.5
Bay of Biscay	3.3 ± 3.5	14.7 ± 4.8	0.7 ± 0.7	3.4 ± 1.6
Atlantic Subtropics	2.2 ± 1.9	0.1 ± 0.1	0.7 ± 0.5	1.3 ± 1.2
Western Mediterranean	10.4 ± 5.4	1.5 ± 3.2	23.2 ± 2.8	4.0 ± 4.7
Eastern Mediterranean	0.0 ± 0.0	0.0 ± 0.0	0.1 ± 0.1	0.0 ± 0.0
Artic	0.0 ± 0.0	0.6 ± 0.4	0.0 ± 0.0	0.0 ± 0.0
Other Seas	0.0 ± 0.0	0.0 ± 0.0	0.0 ± 0.0	0.2 ± 0.1

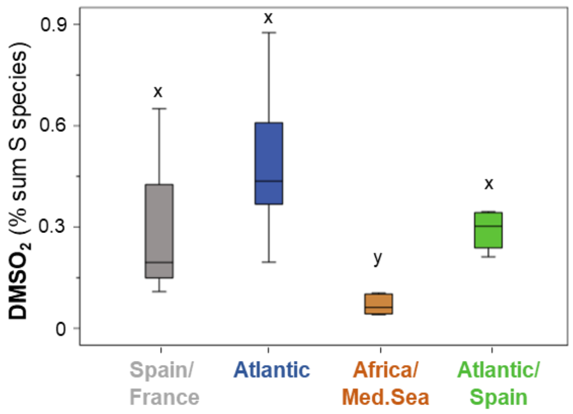

Supplementary Figure 6. Variability of $DMSO_2$ proportions (% sum S species) in precipitation across different moisture source clusters. Different letters (x, y) denote significance levels based on Mann-Whitney-U test, $p<0.05$.

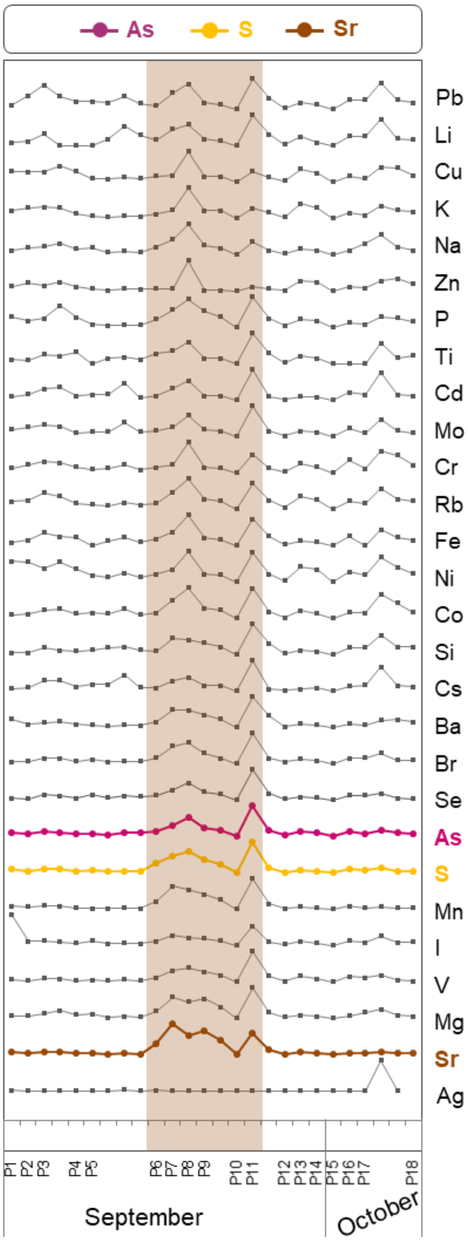

Supplementary Figure 7. Variability in total concentrations of all measured elements in precipitation collected during 2019 campaign, including arsenic (As; pink), sulfur (S; yellow) and strontium (Sr; brown). To remove the influence of high elemental deposition between P6 and P11 (shaded in light brown), Sr was determined as the most suitable element for normalization as it was the only element that showed high elemental deposition solely during this period. Figure adapted from Breuninger et al.[1]

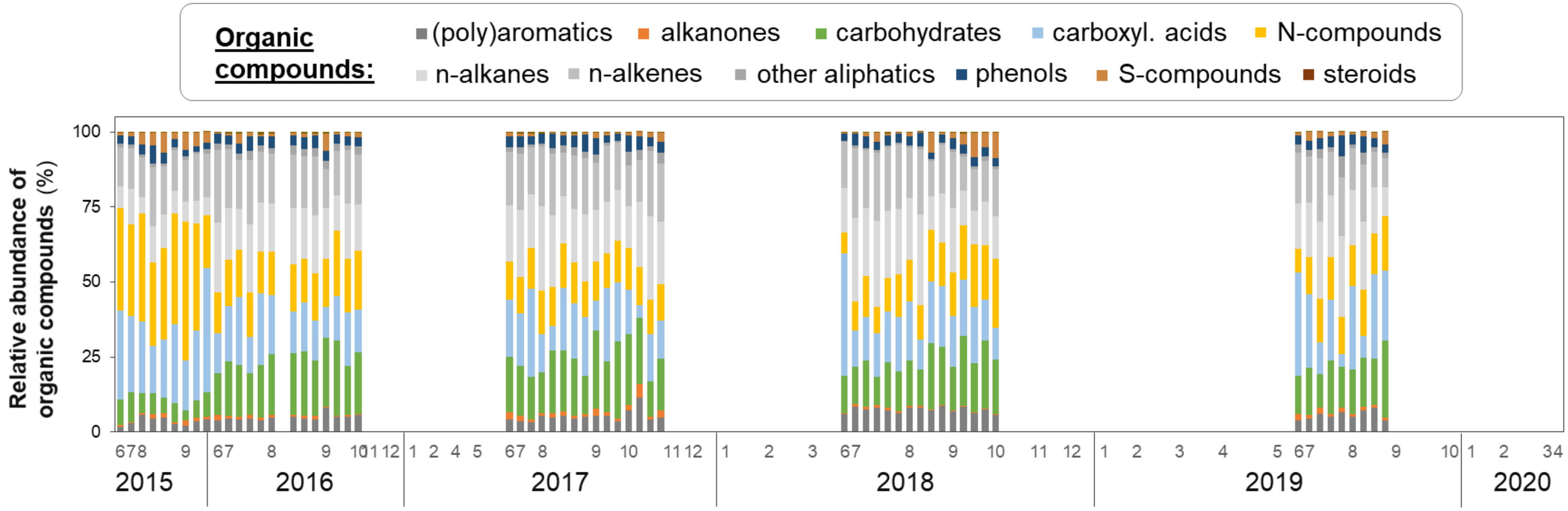

Supplementary Figure 8. Temporal variability of organic compound groups identified by Py-GC/MS in the analysed aerosol time series (2015-2020). Presented compound data (Y-axis) are expressed as relative abundance, i.e., % of sum of peak areas of all organic compounds identified by Py-GC/MS. The X-axis shows the sampling months in which the aerosol samples were collected.

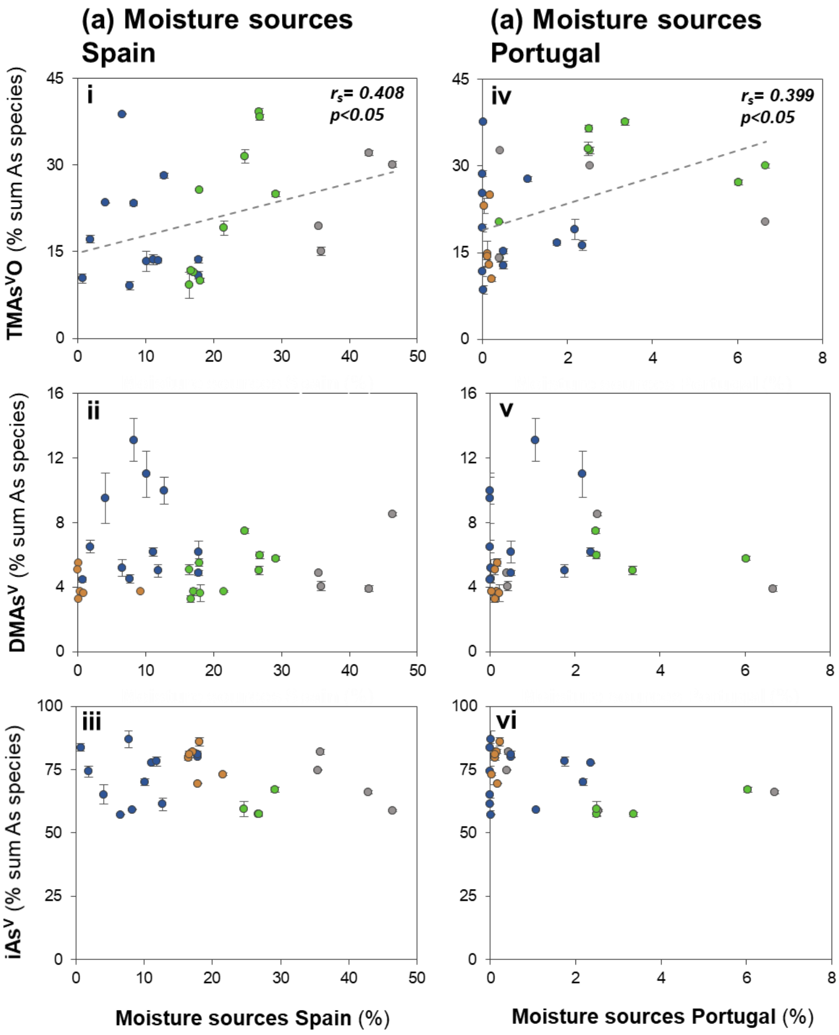

Supplementary Figure 9. As species proportions as a function of moisture sources coming from Spain (panel a) and Portugal (panel b) in (sub)event precipitation samples. All data points of $TMAs^VO$ (top panels i and iv), $DMAs^V$ (middle panels ii and v), and inorganic As (iAs, bottom panels iii and vi) proportions (% sum As species) are colour coded according to moisture source clusters. Indicated correlation coefficients in panels i and iv are significant ($p<0.05$, $n=26$), and the error bars represent the uncertainty of quantification by HPLC-ICP-MS/MS in duplicate, with invisible error bars indicating standard deviations within the symbol.

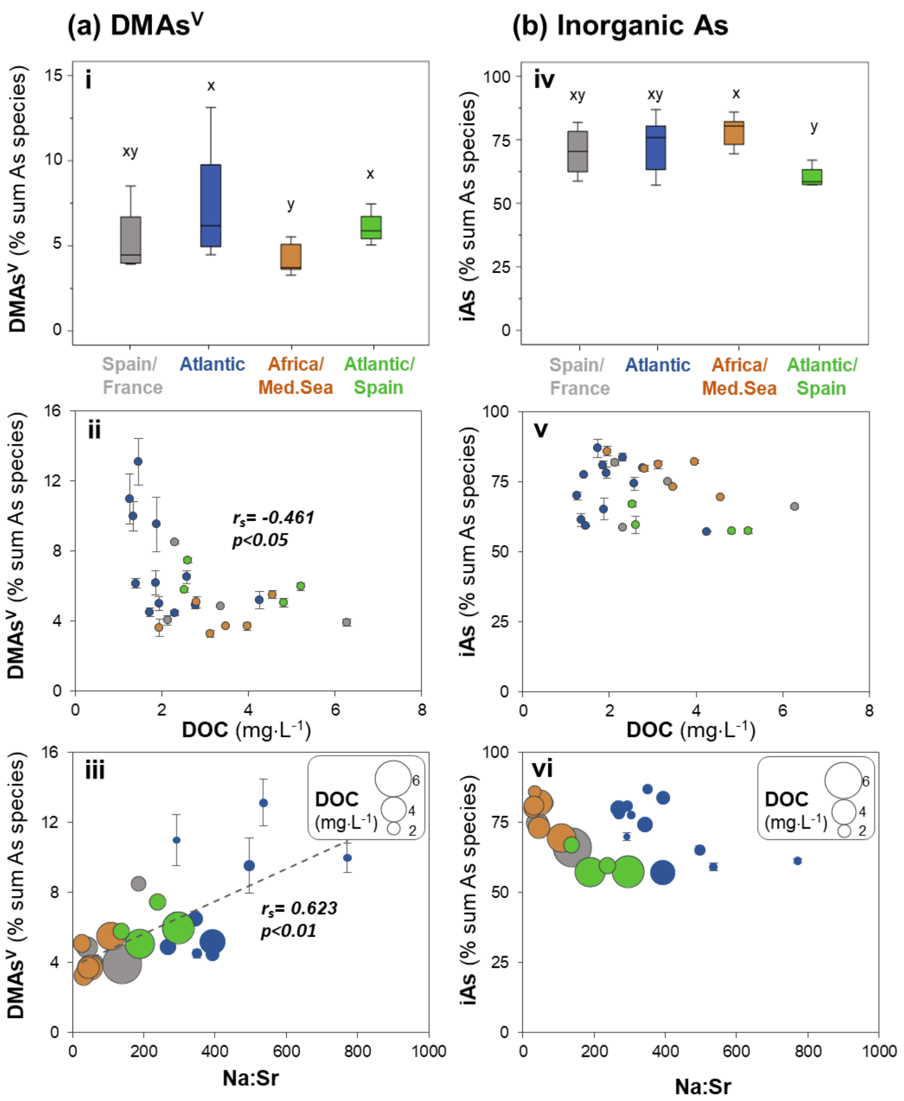

Supplementary Figure 10. Relationships between DMAs^V (panel a) and inorganic As (panel b) in precipitation (sub)events and contributing moisture sources, dissolved organic carbon (DOC) and the sea salt proxy (Na:Sr). The top panels show the variability of DMAs^V (panel i) and inorganic As (panel iv) proportions (% sum As species) in the clusters 1-4 defined by different contributing moisture sources. The boxplots show the interquartile range, representing the middle 50% of the data, which fall between the upper quartile (75% data below that score) and the lower quartile (less than 25% below that score). The whiskers refer to the 5th/95th percentiles. The middle and bottom panels show the variability of both species proportions as a function of dissolved organic carbon (DMAs^V: panel ii, inorganic As: panel v) and as a function of the sea salt proxy Na:Sr (DMAs^V: panel iii, inorganic As: panel vi). The size of data points corresponds to the DOC concentrations in precipitation samples; All symbols in ii, iii, v, and vi are colour coded according to moisture source clusters. Different letters (x, y) in i and iv denote significance levels based on Mann-Whitney-U test ($p<0.05$), i.e., for all clusters with the same letter, the difference between species proportion is not statistically significant. Indicated correlation coefficients in panels ii and iii are significant ($n=26$, $p<0.05$) and the error bars in panels ii, iii, v, and vi represent the uncertainty of quantification by HPLC-ICP-MS/MS in duplicate, with invisible error bars indicating standard deviations within the symbol.

Supplementary Note 1: Principle component analysis of precipitation samples

In the main manuscript, we based our interpretation of the origin of As species in precipitation on moisture sources, indicating significant differences in As species proportions between the 4 identified clusters (Mann-Whitney test), as well as bivariate correlations between As species proportions and chemical source proxies (i.e., methane sulfonic acid -MSA-, Na normalized by Sr -Na:Sr-, sulfur isotopic composition -$\delta^{34}S$-, and dissolved organic carbon concentrations -DOC-). In addition, we performed a principal component analysis (PCA) using the precipitation dataset, after converting data to Z-scores (average=0, variance=1). Only PCs with eigenvalues >1 were selected and extracted using a Varimax rotated solution. The PCA output, with the first four principal components (PC1-PC4) explaining 76% of the total variability, are shown in Supplementary Figure 11. $DMAs^V$ proportions were only found on PC1's positive side (r^2= 0.552) together with the proportion of moisture sources from the Atlantic and Bay of Biscay, and the two marine chemical proxies, i.e., MSA proportions and Na:Sr (Supplementary Figure 11a). $TMAs^VO$ proportions had positive but very low loadings on PC1 (r^2= 0.388; not significant) and plotted more significantly on the negative side of PC3 (Supplementary Figure 11b; described below). Positive scores on PC1 thus represent (sub)event precipitation samples with higher proportions of $DMAs^V$ and marine moisture sources. The samples with positive scores on PC1 included all samples from cluster 2 (Atlantic) and cluster 4 (Atlantic/Spain) (Supplementary Figure 11c), for which significantly higher proportions of $DMAs^V$ were found in comparison to clusters 1 and 3 (Mann-Whitney test; see section "$DMAs^V$ has a dominant marine origin" and Fig. 5a in the manuscript).

$TMAs^VO$ proportions plotted significantly on the negative side of PC3 (r^2= 0.967) together with moisture sources from Portugal, "other seas" and Spain as well as DOC concentrations (Supplementary Figure 11b). Negative loadings on PC3 were also found, although less significant (r^2 between 0.232-0.644), for $DMAs^V$ and indicators for marine moisture source (i.e., Na:Sr, MSA and moisture sources from the Atlantic). Therefore, the negative side of PC3 represents a mixture of land and marine sources and/or coastal source regions that are associated with high $TMAs^vO$ proportions. The samples with negative scores on PC3 included all samples from cluster 4, for which we reported significantly higher proportions of $TMAs^VO$ compared to clusters 1-3 (Mann-Whitney test; see section "$TMAs^VO$ has mixed terrestrial and marine biogenic sources with likely important coastal contributions" and Fig. 4a in the manuscript). Apart from cluster 4, a few samples from clusters 1, 2, and 3 plotted on the negative side of PC3, in line with what we observed when correlating $TMAs^VO$ with DOC and Na:Sr (Fig. 4b and 4c in main manuscript).

Inorganic As (iAs) proportions plotted on the positive side of PC3 (r^2= 0.965), in contrast to other As species or chemical proxies. Positive loadings on PC3 were also found for moisture sources coming from Ireland and other land areas, of which the latter plotted more strongly on the positive side of PC4 (r^2= 0.607-0.648 on PC3 vs 0.659-0.770 on PC4) together with moisture sources from the Arctic region and the UK.

Altogether, the PCA output confirms the clustering of the precipitation (sub)event samples based on the moisture source data and the relationships between As species and specific moisture source regions and/or chemical source proxies as presented in the main manuscript.

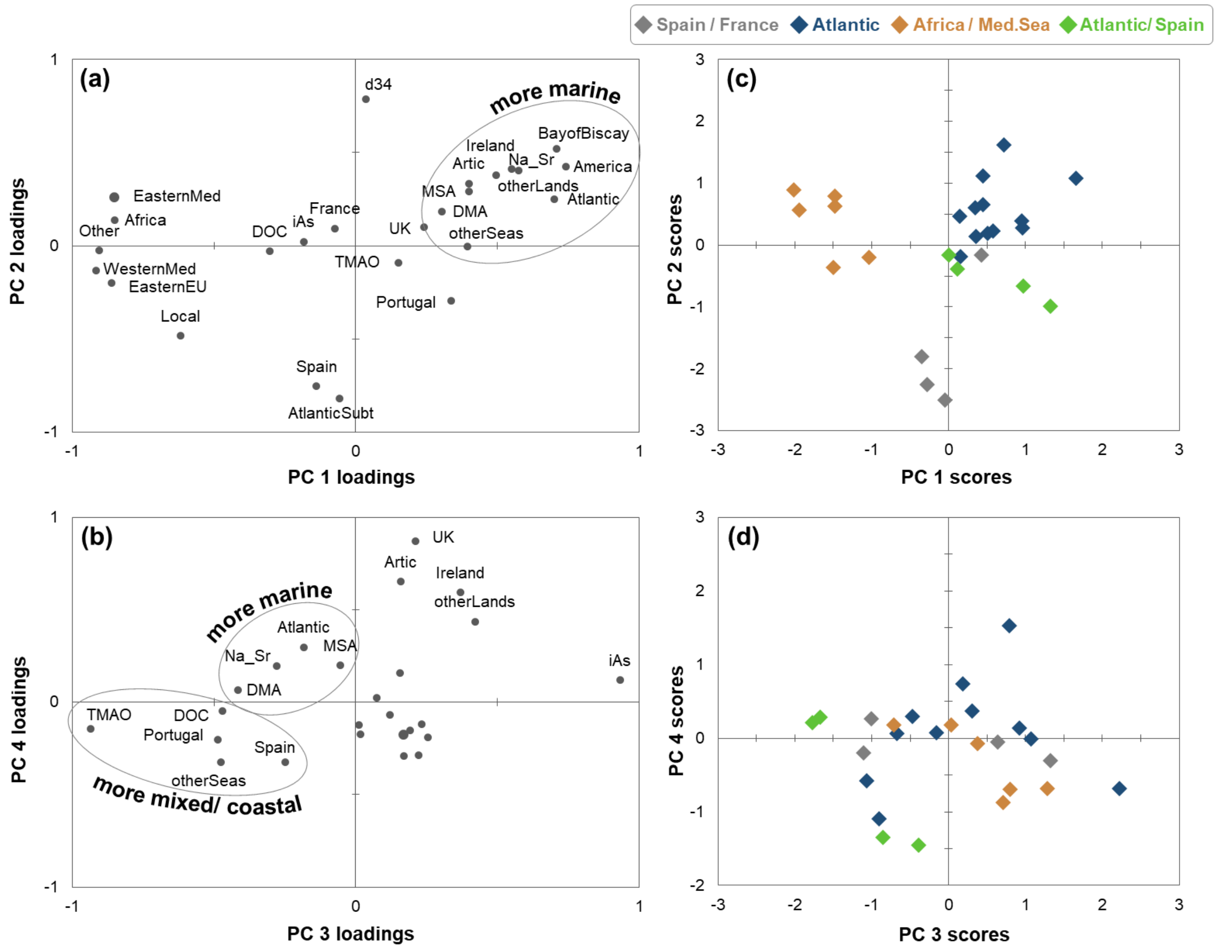

Supplementary Figure 11. Loading plots (panels a, b) and score plots (panels c, d) for principal components (PCs) 1–4 resulting from the principal component analysis performed with the element concentrations in precipitation samples from the 2019 campaign. Four PCs were retained, i.e., PC1: 41%, PC2: 19%, PC3: 9%, PC4: 7% of total variance. Samples scores on PC1-4 shown in panels (c) and (d) are colour coded according to the clusters 1-4 defined by different contributing moisture sources.

Supplementary Table 4. Correlation coefficients between the abundance of (sub-)groups of organic compounds identified by Py-GC/MS and the proportions of species (% sum As species) in different aerosol extracts. Shown As species proportions include iAs (sum of As^{III}, As^{V}), methylated As (sum of $TMAs^{V}O$, $DMAs^{V}$, $MMAs^{V}$), and individual methylAs species. Positive and negative correlations (determined using Spearman correlation coefficient, n=63) are highlighted in blue and orange, respectively. Different significance levels are indicated by colour strength: ***p*<0.01 (dark blue/orange), **p*<0.05 (light blue/orange).

		(Poly)-aromatics	Alkan-ones	Carbo-hydrates	Carboxylic acids	N-comp.	Alkanes	Alkenes	Other aliphatics	Phenols	S-comp.	Steroids
**Water extract**	**iAs**	-0.192	**0.322***	**-0.312***	-0.032	-0.174	0.239	0.227	0.227	0.168	-0.092	**-0.291***
	**methly. As**	0.192	**-0.322***	**0.312***	0.032	0.174	-0.239	-0.227	-0.227	-0.168	0.092	**0.291***
	**$TMAs^{V}O$**	0.147	**-0.326***	**0.316***	0.061	0.224	**-0.308***	**-0.277***	-0.232	-0.215	0.133	**0.276***
	**$DMAs^{V}$**	**0.333****	**-0.309***	**0.265***	-0.045	0.042	-0.021	-0.061	-0.202	-0.040	-0.062	**0.278***
	**$MMAs^{V}$**	-0.070	0.146	0.008	0.039	**-0.274***	**0.296***	0.242	0.162	**0.378****	**-0.338****	-0.028
**1% HNO_3 extract**	**iAs**	-0.101	**0.452****	**-0.353****	-0.149	**-0.276***	**0.422****	**0.374****	**0.341****	**0.459****	-0.074	-0.155
	**methly. As**	0.119	**-0.440****	**0.334****	0.121	**0.258***	**-0.383****	**-0.364****	**-0.347****	**-0.449****	0.053	0.103
	**$TMAs^{V}O$**	0.060	**-0.479****	**0.453****	0.098	**0.368****	**-0.492****	**-0.443****	-0.145	**-0.430****	0.127	0.209
	**$DMAs^{V}$**	0.163	**-0.341***	**0.341***	-0.053	0.038	-0.059	0.031	**-0.282***	-0.145	-0.188	**0.313***
	**$MMAs^{V}$**	0.265	-0.139	-0.119	-0.179	-0.179	**0.382****	0.242	-0.197	**0.343***	-0.155	0.014

Supplementary Discussion 3: Photochemical Experiments

Motivation. We performed photochemical experiments to test the potential abiotic formation of pentavalent methylated As species from inorganic As in the presence of low molecular organic acids. In a previous study, Guo et al.[15] reported the photochemical alkylation of inorganic As to volatile methylated As species in the presence of low molecular weight organic acids under UV-C light (i.e., ultraviolet radiation with shorter wavelengths: 100-280 nm). The authors found high methylation rates for solutions containing arsenite ($As^{III}O_4^{3-}$) and mixtures of acetic acid and formic acid, with As-to-organic acid molar ratios of 1-to-10^7. When using arsenite ($As^{V}O_4^{3-}$), no significant generation of volatile As species was observed[15].

However, UV-C radiation is largely absorbed and/or reflected in the stratospheric ozone layer and therefore not very representative for atmospheric conditions in the troposphere[16]. To test potential photochemical formation of methylated As species under more realistic atmospheric conditions, we irradiated aqueous solutions of $As^{III}O_4^{3-}$, organic acids (formic, acetic and oxalic acid), and dissolved organic matter (as photosensitizer) over a pH range of 2 to 4 using more environmentally relevant UV radiation (290-345 nm).

Experimental details. All experiments were conducted with a photochemical reactor (Southern New England Ultraviolet Company) equipped with a cooler, a motorized turntable, and 6 UV light bulbs (Southern New England Ultraviolet Co., RPR 3000A, peak emission at 310 nm). Throughout all experiments, the solution temperature ranged from 29 to 31 °C. Aqueous solutions were irradiated in 20 mL scintillation vials (DWK Life Sciences Wheaton™, Thermo Scientific) closed with screw caps with integrated PTFE septum.

To ensure detection of all As species of interest, we increased 100 times the concentration of each solution component while keeping their relative ratios the same as typical environmental samples. Previously reported As concentrations in rainwater are ~0.050 $\mu g \cdot L^{-1}$,[3, 4] while molar ratios between As and organic acids are ~1:10^4 [17, 18, 19, 20, 21] in rain and cloud water[17, 18, 19, 20, 21]. Accordingly, for tested $As^{III}O_4^{3-}$ concentrations of 5 $\mu g(As) \cdot L^{-1}$ (0.067 $\mu mol(As) \cdot L^{-1}$), we used 667 $\mu mol \cdot L^{-1}$ of organic acids (i.e., 7.5 $mg(C) \cdot L^{-1}$ of formic acid and 15 $mg(C) \cdot L^{-1}$ for acetic and oxalic acid, respectively). Natural organic matter (SRNOM, 1R101N, International Humic Substance Society) was also added as natural sensitizer with concentrations of 40 $mg(C) \cdot L^{-1}$, approximately two orders of magnitude higher than previously reported humic like substances in rainwater (i.e., 0.1-0.5 $mg(C) \cdot L^{-1}$)[22]. The use of a sensitizer is required because neither $As^{III}O_4^{3-}$ nor the tested acids absorb light >280 nm[23, 24, 25, 26]. These solutions were prepared using either ultrapure water or rainwater as the solvent.

At the beginning (t0) and various hourly time points (up to 6 h), we collected an aliquot (500 µL) for quantification of As species (i.e., $As^{III}O_4^{3-}$, $MMAs^V$, $DMAs^V$, $TMAs^VO$ and $As^VO_4^{3-}$) via HPLC-ICP-MS/MS (detailed description in Supplementary Discussion 2). All experiments were performed in duplicate.

Results. The first experiments consisted of irradiation of:

- Solutions of As^{III} (5 $\mu g(As) \cdot L^{-1}$), formic acid (7.5 $mg(C) \cdot L^{-1}$), acetic acid (15 $mg(C) \cdot L^{-1}$), oxalic acid (15 $mg(C) \cdot L^{-1}$), natural organic matter (40 $mg(C) \cdot L^{-1}$) in ultrapure water at pH 2 (Supplementary Figure 12a) and pH 4 (Supplementary Figure 12b)
- Solutions of As^{III} (5 $\mu g \cdot L^{-1}$), oxalic acid (15 $mg(C) \cdot L^{-1}$), natural organic matter (40 $mg(C) \cdot L^{-1}$) in ultrapure water at pH 2 (Supplementary Figure 12c) and pH 4 (Supplementary Figure 12d)
- Solutions of As^{III} (5 $\mu g(As) \cdot L^{-1}$), natural organic matter (40 $mg(C) \cdot L^{-1}$) in ultrapure water at pH 2 (Supplementary Figure 12e) and pH 4 (Supplementary Figure 12f)

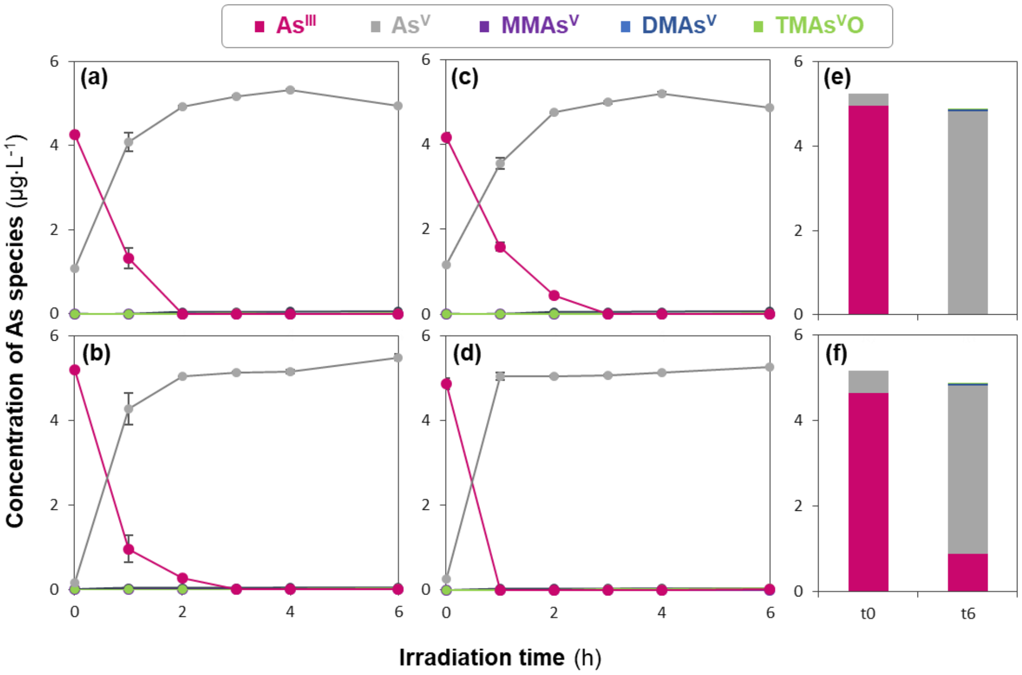

Supplementary Figure 12. Transformation of As species concentrations during irradiation experiments performed in ultrapure water over 6 h. Panel a and b show As species transformation for solutions containing $As^{III}O_4^{3-}$, formic acid, acetic acid, oxalic acid, and natural organic matter at pH 2 **(a)** and pH 4 **(b)**. Panel c and d show As species transformation for solutions containing $As^{III}O_4^{3-}$, oxalic acid, and natural organic matter at pH 2 **(c)** and pH 4 **(d)**. Panel e and f show As species concentrations in the beginning (t0) and the end of irradiation for solutions containing As^{III} and natural organic matter at pH 2 **(e)** and pH 4 **(f)**. The error bars represent the standard deviation values resulting from two independent replicates.

Irradiation experiments performed at pH 2 and 4 in ultrapure water (Supplementary Figure 12) demonstrated the rapid and quantitative oxidation of spiked As^{III} to As^{V} for different mixtures of organic acids and natural organic matter (Supplementary Figure 12a-d) and solutions only containing natural organic matter (Supplementary Figure 12e-f) within the first two hours. During the irradiation period, we did not detect any methylated As species.

In a following test, two different rainwater samples collected at Pic du Midi Observatory (previously stored at 4°C) were irradiated using similar mixtures described above:

- Solutions of As^{III} (5 µg(As)·L^{-1}), formic acid (7.5 mg(C)·L^{-1}), acetic acid (15 mg(C)·L^{-1}), oxalic acid (15 mg(C)·L^{-1}), natural organic matter (40 mg(C)·L^{-1}) in two different rainwater samples
- Solutions of As^{III} (5 µg(As)·L^{-1}) in two different rainwater samples

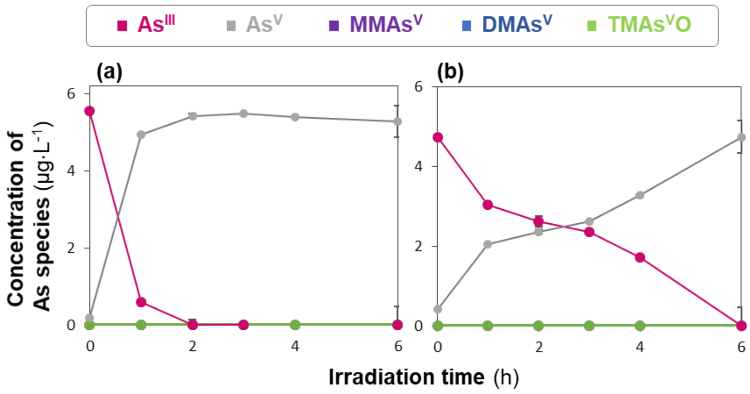

Supplementary Figure 13. Transformation of As species concentrations during irradiation experiments performed in two different rainwater samples collected at Pic du Midi Observatory water over 6 h. Panel **(a)** shows the As species transformation for solutions containing As^{III}, formic acid, acetic acid, oxalic acid, and natural organic matter. Panel **(b)** shows the As species transformation for solutions containing As^{III} and rainwater. The error bars represent the standard deviation values resulting from two independent replicates (i.e., rainwater samples).

Similar to irradiation experiments performed in ultrapure water, solutions prepared in rainwater demonstrated rapid and quantitative oxidation of spiked As^{III} to As^{V}, with no detected formation of methylated As species during the irradiation period. Irradiation of solutions containing As^{III} and rainwater in the absence of organic acids and natural organic matter (Supplementary Figure 13b) showed slower oxidation rates compared to other experiments, in agreement with its lower DOC (and consequently reactive intermediate concentrations) as compared to solution spiked with SRNOM (i.e., 1.2 vs. 40 mg(C)·L^{-1}).

Overall, our results suggest no significant abiotic formation of methylated As species from As^{III} under tested atmospheric conditions.

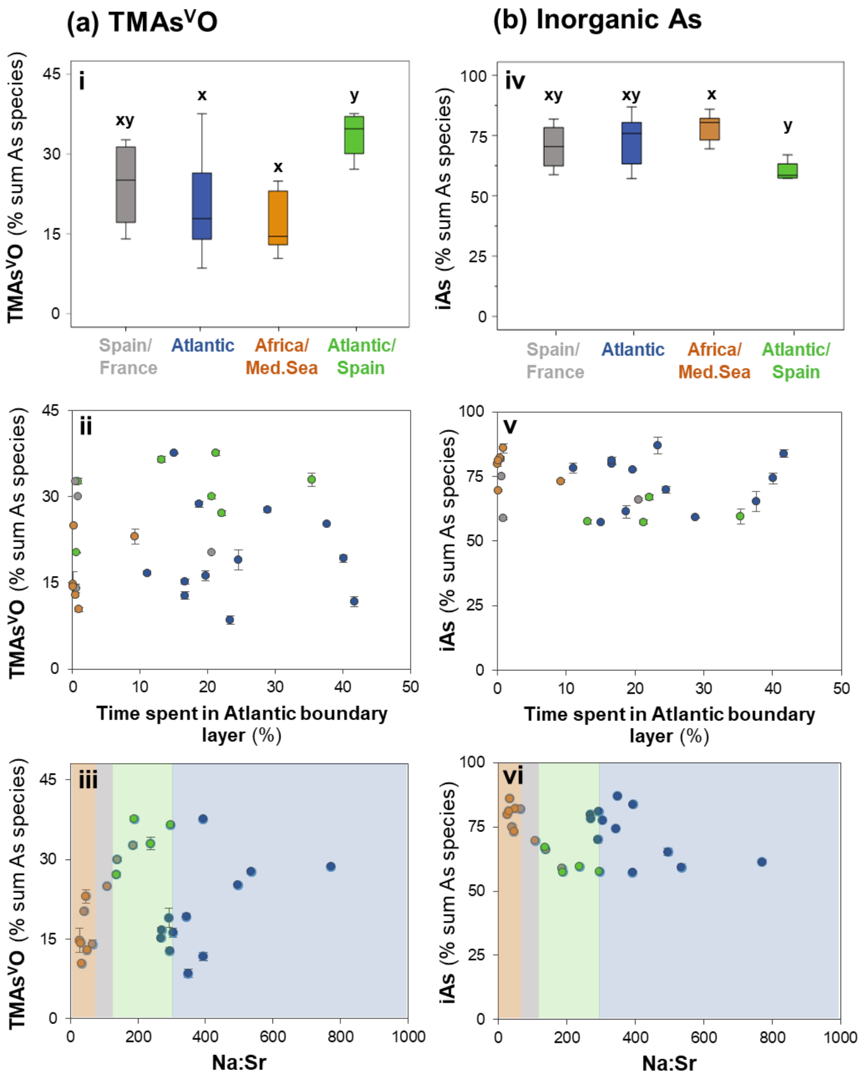

Supplementary Figure 14. Relationships of TMAs^{V}O (panel a) and inorganic As (panel b) with contributing moisture sources, the time spent in the Atlantic boundary layer and the sea salt proxy (Na:Sr) in precipitation (sub)events. The top panels show the variability of TMAs^{V}O (panel i) and inorganic As (panel iv) proportions (% sum As species) in the clusters 1-4 defined by different contributing moisture sources. The boxplots show the interquartile range, representing the middle 50% of the data, which fall between the upper quartile (75% data below that score) and the lower quartile (less than 25% below that score). The whiskers refer to the 5th/95th percentiles. The middle and bottom panels show the variability of both species proportions as a function of the time spend in the Atlantic boundary layer (TMAs^{V}O: panel ii, inorganic As: panel v) and as a function of the sea salt proxy Na:Sr (TMAs^{V}O: panel iii, inorganic As: panel vi). All symbols in panels ii, iii, v, and vi are colour coded according to moisture source clusters. Different letters (x, y) in i and iv denote significance levels based on Mann-Whitney-U test ($p<0.05$), i.e., for all clusters with the same letter, the difference between species proportion is not statistically significant. The error bars in panels ii, iii, v, and vi represent the uncertainty of quantification by HPLC-ICP-MS/MS in duplicate, with invisible error bars indicating standard deviations within the symbol.

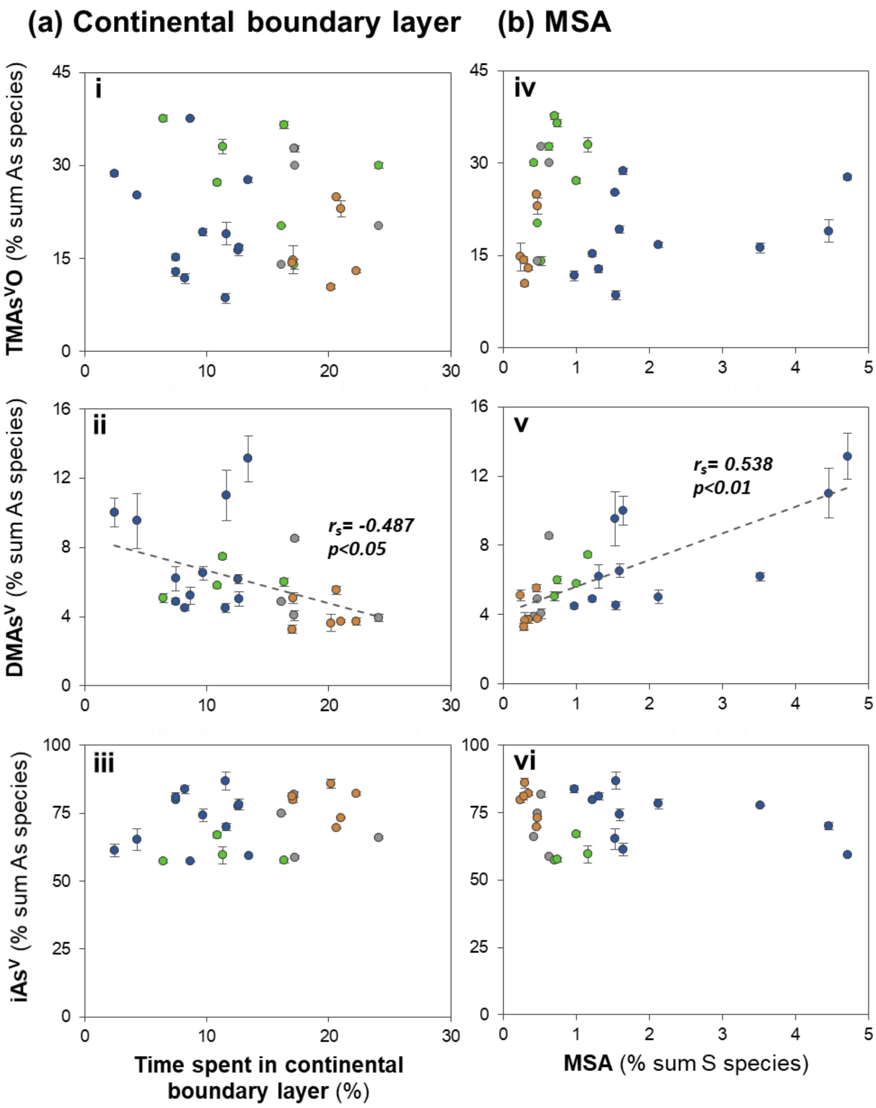

Supplementary Figure 15. As species proportions as a function of the time spend in the continental boundary layer (panel a) and proportions of methane sulfonic acid (MSA; panel b) in (sub) event precipitation samples. All data points of $TMAs^{V}O$ (top panels i and iv), $DMAs^{V}$ (middle panels ii and v), and inorganic As (iAs, bottom panels iii and vi) proportions (% sum As species) are colour coded according to moisture source clusters. Indicated correlation coefficients in panels ii and v are significant ($p<0.05$, n=26. The error bars represent the uncertainty of quantification by HPLC-ICP-MS/MS in duplicate, with invisible error bars indicating standard deviations within the symbol.

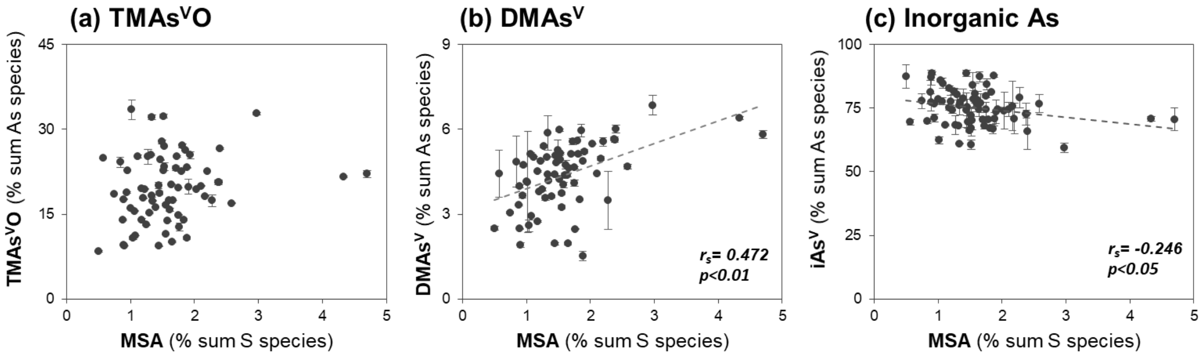

Supplementary Figure 16. Proportions of $TMAs^VO$, $DMAs^V$ and inorganic As (iAs) as a function of the proportions of methane sulfonic acid (MSA) in the aerosol water extracts of the 2015-2020 time series. Indicated correlation coefficients in panels b and c are significant ($p<0.05$, $n=70$), and the error bars represent the uncertainty of quantification by HPLC-ICP-MS/MS in duplicate, with invisible error bars indicating standard deviations within the symbol.

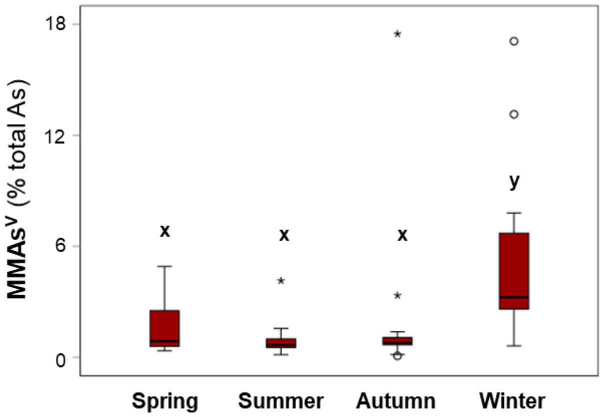

Supplementary Figure 17. Variability of $MMAs^V$ proportions in the 1% HNO_3 aerosol extract in different seasons. As species proportions (% total As in extract) are shown for spring (March-May; n=9), summer (June-August; n=35), autumn (September-October; n=27), and winter (December-February; n=15) months during the 2015-2020 aerosol series. The boxplots show the interquartile range, representing the middle 50% of the data, which fall between the upper quartile (75% data below that score) and the lower quartile (less than 25% below that score). The whiskers refer to the 5th/95th percentiles. Different letters (x, y) denote significance levels based on Mann-Whitney-U test ($p<0.05$), i.e., for all clusters with the same letter, the difference between species proportion is not statistically significant.

Supplementary Discussion 4: Atmospheric deposition flux of methylated As and comparison to in-soil methylation rates

To investigate the potential impact of atmospheric inputs of methylated As to soil systems, we compared deposition fluxes of methylated As species based on our precipitation data collected during the high-resolution campaign with previously reported As methylation rates in soils. The atmospheric deposition flux ($\mu g \cdot L^{-1} \cdot d^{-1}$) of methylated As species (sum of $TMAs^{V}O$, $DMAs^{V}$, $MMAs^{V}$) was calculated using the weighted sample volume and duration of individual sub-events. The wet deposition flux ranged between 0.01 and 3.38 $\mu g \cdot L^{-1} \cdot d^{-1}$ with an average of 0.5±0.8 $\mu g \cdot L^{-1} \cdot d^{-1}$.

We compared this deposition flux to in-soil production rates based on previous published measurements of methylated As species determined in pore water of natural soils (pot experiments with paddy and arable soils from Bangladesh, China, UK and USA) with varying contamination levels (contamination by irrigation, mining, high geogenic background or no contamination; Supplementary Table 5)[57, 59]. Specific methylation rates were calculated based on the maximum observed methylation efficiency, which describes the percentage of substrate (i.e., initial As^{III} concentration) converted to methylated As species within specific incubation times[27]. As^{III} has been identified as the primary substrate for As methylation for various microbial strains[27]. The two considered studies solely reported the formation of $MMAs^{V}$ and $DMAs^{V}$. Although it should be noted that the analysis of $TMAs^{V}O$ was not indicated.

Supplementary Table 5. Estimated in-soil methylation rates of different paddy and arable soils from Bangladesh, China, UK and USA with varying contamination sources. Methylation efficiencies and rates were calculated based on reported concentrations of As^{III} (substrate) and methylated As species in soil pore waters[28, 29].

**Soil classification** *(region)*	**Contamination source**	**Initial substrate (As^{III}) concentration** ($\mu g \cdot L^{-1}$)	**Maximum methylation efficiency[a]** (%)	**Incubation time** (d)	**Methylation rate** ($\mu g \cdot L^{-1} \cdot d^{-1}$)
paddy soil *(Bangladesh)*	Irrigation	50	14	49	0.14
paddy soil *(Bangladesh)*	Irrigation	90	2.2	49	0.04
paddy soil *(China)*	Mining	180	0.5	49	0.02
paddy soil *(China)*	Geogenic	160	0.3	49	0.01
arable *(UK)*	None	4	2.6	49	0.002
arable *(USA)*	None	2.5	9.5	49	0.005
Arable *(UK)*	None	38	47	44	0.41

[a]Maximum methylation efficiencies were calculated as percentage of substrate (i.e., initial As^{III} concentration) converted to methylated As species.

The estimated maximum methylation efficiencies range between 0.5-47%, which is similar to previously reported methylation efficiencies in pure and mixed cultures[27, 30, 31]. Methylation efficiencies in natural soils are generally expected to be lower compared to tested cultures due to the abundance and diversity of As methylating microbes across different soils as well as other biotic and abiotic factors[28]. We specifically used methylation rates based on measurements in natural soils to enable the comparison of environmentally relevant conditions. The estimated soil methylation rates, considering methylation efficiencies and incubation time of listed studies, range between 0.002 and 0.41 $\mu g \cdot L^{-1} \cdot d^{-1}$ (average: 0.09±0.15 $\mu g \cdot L^{-1} \cdot d^{-1}$), which is lower than the average atmospheric inputs we estimated.

Supplementary Table 6. Average recoveries (%) and error (%) obtained for measured elements in two certified reference materials (CRMs) which were analysed with atmospheric deposition samples. The CRMs were analysed after their dilution (d10 or d100) in the corresponding sample matrix, i.e., 1% HNO_3 for the precipitation, cloud water and aerosol water extracts samples, and 16-26% HNO_3 for the aerosol digests. N gives the number of analysed replicates for each CRM. Table previously published in Breuninger et al.[1].

		**Precipitation & Cloud water** (1% HNO_3)				**Aerosol Extract** (16-26% HNO_3)			
	**Certified element concentration** (µg·L^{-1})	**Recoveries**[a] (%)	**Error**[b] (%)	**Used Dilution**	**N**	**Recoveries**[a] (%)	**Error**[b] (%)	**Used Dilution**	**N**
**CRM NIST 1643f, Trace Elements in Water**									
**Li**	16.6 ± 0.4	**103** ± 9	**3**	d10, d100	4	**99.5** ± 4	**-0.5**	d10, d100	6
**Na**	18830 ± 250	**102** ± 2	**2**	d10, d100	4	**98** ± 2	**-2**	d10, d100	12
**Mg**	7454 ± 60	**103** ± 2	**3**	d10, d100	4	**102.7** ± 1.1	**2.7**	d10, d100	12
**Al**	133.8 ± 1.2	±			4	**105** ± 3	**5**	d10, d100	12
**K**	1933 ± 9	**100.4** ± 6.7	**0.4**	d10	4	**98.5** ± 0.7	**-1.5**	d10, d100	12
**V**	36.1 ± 0.3	**105** ± 0	**5**	d10	4	**103** ± 2	**3**	d10, d100	6
**Cr**	18.5 ± 0.1	**101** ± 8	**1**	d10, d100	4	**105** ± 3	**5**	d10, d100	6
**Mn**	37.1 ± 0.6	**103** ± 4	**3**	d10, d100	4	**98.7** ± 2.3	**-1.3**	d10, d100	6
**Fe**	93.4 ± 0.8	**105** ± 3	**5**	d10	4	**101.8** ± 0.5	**1.8**	d10, d100	12
**Co**	25.3 ± 0.2	**98** ± 4	**-2**	d10, d100	4	**103.0** ± 0.1	**3.0**	d10, d100	6
**Ni**	59.8 ± 1.4	**99.6** ± 3.0	**-0.4**	d10, d100	4	**101.9** ± 0.4	**1.9**	d10, d100	6
**Cu**	21.7 ± 0.7	**102** ± 7	**2**	d10, d100	4	**102.3** ± 1.6	**2.3**	d10, d100	6
**Zn**	74.4 ± 1.7	**102** ± 4	**2**	d10	4	**105** ± 3	**5**	d10	6
**As**	57.4 ± 0.4	**99** ± 0.7	**-1**	d10, d100	4	**103.7** ± 0.7	**3.7**	d10, d100	6
**^{78}Se**	11.7 ± 0.1	**99** ± 1	**-1**	d10, d100	4	**102.9** ± 0.5	**2.9**	d10, d100	6
**^{80}Se**	11.7 ± 0.1	**99.2** ± 0.4	**-0.8**	d10, d100	4	**101.2** ± 0.1	**1.2**	d10, d100	6
**Rb**	12.6 ± 0.1	**97** ± 7	**-3**	d10	4	**105.5** ± 1.0	**5.5**	d10, d100	6
**Sr**	314 ± 19	**101** ± 5	**1**	d10, d100	4	**103.2** ± 1.4	**3.2**	d10, d100	6
**Mo**	115.3 ± 1.7	**99** ± 5	**-1**	d10, d100	4	**103.4** ± 0.4	**3.4**	d10, d100	6
**Ag**	1.0 ± 0.0	**97** ± 7	**-3**	d10	4	**107** ± 4	**7**	d10	6
**Cd**	5.9 ± 0.1	**102** ± 4	**2**	d10, d100	4	**102** ± 3	**2**	d10, d100	6
**Ba**	518.2 ± 7	**102** ± 2	**2**	d10, d100	4	**102.2** ± 0.2	**2.2**	d10, d100	6
**Pb**	18.5 ± 0.1	**99.7** ± 1.7	**-0.3**	d10	4	**103.1** ± 1.5	**3.1**	d10, d100	6
**CRM TMDA 51.2, Trace Elements in Surface Water (Lake Ontario)**									
**Al**	96 ± 19					**99.2** ± 7.8	**-0.8**	d10	6
**V**	48 ± 8	**102** ± 2	**2**	d10, d100	4	**102.7** ± 0.5	**2.7**	d10, d100	6
**Cr**	63 ± 7	**97** ± 5	**-3**	d10, d100	4	**103.8** ± 1.3	**3.8**	d10, d100	6
**Mn**	82 ± 10	**98** ± 2	**-2**	d10, d100	4	**102.0** ± 1.0	**2.0**	d10, d100	6
**Fe**	111 ± 26	**108** ± 6	**8**	d10	4	**103** ± 3	**3**		6
**Co**	72 ± 6	**94** ± 8	**-6**	d10, d100	4	**104.8** ± 1.0	**4.8**	d10, d100	6
**Ni**	67 ± 7	**97.7** ± 0.9	**-2.3**	d10, d100	4	**104.9** ± 0.3	**4.9**	d10, d100	6
**Cu**	91 ± 10	**99** ± 3	**-1**	d10, d100	4	**104.4** ± 1.2	**4.4**	d10, d100	6
**Zn**	106 ± 15	**102** ± 3	**2**	d10	4	**105.6** ± 0.4	**5.6**	d10, d100	6
**As**	15 ± 3	**96.0** ± 1.2	**-4**	d10, d100	4	**103.1** ± 0.9	**3.1**	d10, d100	6
**^{78}Se**	12 ± 3	**96.7** ± 0.3	**-3.3**	d10, d100	4	**97.6** ± 0.3	**-2.4**	d10, d100	6
**^{80}Se**	12 ± 3	**96.0** ± 1.2	**-4**	d10, d100	4	**98.4** ± 2.0	**-2**	d10, d100	6
**Sr**	121 ± 12	**96** ± 4	**-4**	d10	4	**101.7** ± 0.4	**1.7**	d10, d100	6
**Mo**	59 ± 6	**97** ± 5	**-3**	d10, d100	4	**106.3** ± 1.0	**6.3**	d10, d100	6
**Cd**	25 ± 3	**99.2** ± 1.0	**-0.8**	d10, d100	4	**103** ± 2	**3**	d10, d100	6
**Ba**	73 ± 6	**99** ± 3	**-1**	d10, d100	4	**104.3** ± 0.8	**4.3**	d10, d100	6
**Pb**	73 ± 11	**99** ± 4	**-1**	d10, d100	4	**102.0** ± 0.5	**2.0**	d10, d100	6

[a]Recoveries were calculated as follows: (element concentration measured)/(element concentration certified) x100;
[b]Errors were calculated as follows: [(element concentration measured)-(element concentration certified)] / (element concentration certified) x100

Supplementary Table 7. HPLC-ICP-MS/MS operating conditions for speciation analysis of As.

**LC system**	**Agilent 1260 Infinity II Bio-Inert high performance LC System**
**Column**	Hamilton PRPX-100, 150x2.1mm, 5 µm
**Injection Volume**	20 µL
**Mobile Phase**	5 mmol·L^{-1} Malonic acid (optimized), pH 5.4
**Flow Rate**	0.4-0.5 mL min^{-1}
**ICP-MS/MS system**	**8900 Agilent ICP-MS/MS**
**Configuration**	Quartz torch (2.5 mm id), Ni cones, x-lenses
**RF power**	1550 W
**Spray Chamber**	Scott type, + 2°C
**Nebulizer Gas**	1.10 L min^{-1}
**Make up Gas**	0.10 L min^{-1}
**Cell Gas**	30% of O_2 with 1 ml min^{-1} of H_2
**Acquisition time**	300 ms for ^{75}As,<br>10 ms for Y

Supplementary Method 1: Analysis of organic compounds in aerosol samples by Py-GC/MS

The analysis of organic compounds in aerosols was carried out using a FrontierLab pyrolyzer (equipped with a FrontierLab AS-1020E autosampler) connected to a GC/MS system (Trace 1310 GC, ISQ 7000 MS, Thermoscientific). The experimental conditions and data processing method followed the protocol described by Tolu et al.[32].

Peak identification was done using the software "NIST MS Search 2," which includes the library "NIST/EPA/NIH 2011" and additional spectra from published studies[32]. We identified 105 pyrolysis compounds from 11 classes in the warm season aerosol samples (collected from June-October, 2015-2019). These compounds included, among others carbohydrates, N compounds, and (poly)aromatics. For each compound, we obtained the molecular mass and chemical formula, along with references for the theoretical mass spectra. To facilitate comparison, the relative abundance of each compound was calculated by setting the total identified peak area for each sample to 100%. Individual pyrolysis products were grouped based on similarities in their molecular structure and origin, and these groups were used for further statistical analysis (see defined compound (sub)groups in Supplementary Table 8).

Supplementary Table 8. List of organic compounds identified by Py-GC/MS in aerosol samples, including their formula, molecular mass (M), specific mass fragments with respective references of the mass spectra, and the compound sub-groupings used for the statistical analyses. Table previously published in Breuninger et al.[1]

Name	Formula	M	Specific mass fragments	Ref	Compound sub-groups
**Carbohydrates**					
4,4-Dimethyl-2-cyclopenten-1-one	C7H10O	110	41+67+**95**+110	NIST	(Cyclo)pentenone/-dione
4-Cyclopentene-1,3-dione	C5H4O2	96	26+42+54+68+**96**	NIST	
3-Furaldehyde	C5H4O2	96	39+67+**95**+96	NIST	(alkyl)furans/ -furanones
2(3H)-Furanone, 5-methyl-	C5H6O2	98	27+43+**55**+98	NIST	
2(5H)-Furanone	C4H4O2	84	39+55+84	NIST	
2(5H)-Furanone, 5,5-dimethyl-	C6H8O2	112	43+96+**97**	NIST	
(3H)-Furanone, dihydro-5-methyl-	C5H8O2	100	56+85+100	NIST	
2-Furaldehyde, 5-methyl-	C6H6O2	110	27+53+**110**	NIST	
4-Methyl-5H-furan-2-one	C5H6O2	98	39+40+41+**69**+98	NIST	
2(5H)-Furanone, 3,5,5-trimethyl-	C7H10O2	126	83+111	NIST	
2(3H)-Benzofuranone	C8H6O2	134	**78**+106+134	NIST	
1,4:3,6-Dianhydro-α-d-glucopyranose	C6H8O4	144	29+41+57+**69**	NIST	Levoglucosan or fresh polyaccharides
β-D-Glucopyranose, 1,6-anhydro-	C6H10O5	162	29+57+**60**+73	NIST	
**Carboxylic acids**					
2-Propenoic acid	C3H4O2	72	26+27+45+55+**72**	NIST	Alkanoic acids C3-C12
2-Pentenoic acid, 2-methyl-, (E)-	C6H10O2	114	27+39+41+**69**+114	NIST	
Pinonic acid	C10H16O3	184	69+83+98+114+125	NIST	
Dodecanoic acid	C12H24O2	200	41+43+55+57+60+**73**	NIST	
Tetradecanoic acid	C14H28O2	228	41+43+55+57+60+**73**+129+228	NIST	Alkanoic acids C14-C18
Pentadecanoic acid	C15H30O2	242	41+43+55+57+60+**73**+129+242	NIST	
Hexadecanoic acid	C16H32O2	256	41+**43**+55+57+60+73+129+256	NIST	
Octadecenoic acid	C18H34O2	282	55+69+83+97+123+264+282	NIST	
Octadecanoic acid	C18H36O2	284	41+**43**+55+57+60+73+129+284	NIST	
Alkanoic acid methyl ester			74+87+255+298	NIST	Alkanoic acids >C18
Alkanoic acid C22:0	C22H44O2	332	41+**43**+55+57+60+73+129+332	NIST	
**N-compounds**					
Pyrrole+pyridine	(C4H5N)+ (C5H5N)	(83)+(79)	(50+51+52+**79) + (67)**	NIST	(alkyl)pyrroles/ -pyridines
Pyrrole	C4H5N	67	37+38+**39**+40+**41**+**67**	NIST	
1H-Pyrrole, 2-methyl-	C5H7N	81	**80**+81	NIST	
Pyridine, 3-methyl-	C6H7N	93	39+65+66+92+**93**	NIST	
Pyridine, 2,4,6-trimethyl-	C8H11N	121	79+106+**121**	NIST	
Pyrazole-5-carboxylic acid	C4H4N2O2	112	66+94+95+112	NIST	
1H-Pyrrole-2,5-dione, 1-methyl-	C5H5NO2	111	26+54+83+83+**111**	NIST	
2,5-Pyrrolidinedione (succinimide)	C4H5NO2	99	59+99	NIST	
1H-Pyrazole, 1,3,5-trimethyl-	C6H10N2	110	95+**110**	NIST	
Acetamide	C2H5NO	59	42+43+**44+59**	NIST	Alkylamides
Alkylamide1	unknown		**59+**72	NIST	

Name	Formula	M	Specific mass fragments	Ref	Compound sub-groups
Alkylamide2	unknown		**59+**72	NIST	
Alkylamide3	unknown		**59+**72	NIST	
Docosenamide	C22H43NO	337	**59+72**+240+277+294+320+337	NIST	
Diketopiperazine (DKP) Pro-Pro	C10H14N2O 2	194	70+96+138+166+ **194**	[1, 2]	Proteins
Diketopiperazine (DKP) Pro-Lys-NH3	unknown	208	70, 125, 154, 166, **208**	[1, 2]	
Octadecanenitrile	C18H35N	265	**57**+70+97+110+ 124	NIST	Alkylnitriles
alkanenitrile1	unknown	x	**57**+70+97+110+ 124	NIST	
alkanenitrile2	unknown	x	**57**+70+97+110+ 124	NIST	
alkanenitrile3	unknown	x	**57**+70+97+110+ 124	NIST	
alkanenitrile4	unknown	x	**57**+70+97+110+ 124	NIST	
***n*-alkenes**					
n-C13:1	C13H26	182	56+69+…+182	NIST	C13 (odd n°)
n-C14:1	C14H28	196	56+69+…+196	NIST	C14 to C20 (even n°)
n-C16:1	C16H32	224	56+69+…+224	NIST	
n-C18:1	C18H36	252	56+69+…+252	NIST	
n-C20:1	C20H40	280	56+69+…+280	NIST	
n-C15:1	C15H30	210	56+69+…+210	NIST	C15 to C19 (odd n°)
n-C17:1	C17H34	238	56+69+…+238	NIST	
n-C19:1	C19H38	266	56+69+…+266	NIST	
n-C21:1	C21H42	294	56+69+…+294	NIST	C21 C33 (odd n°)
n-C23:1	C23H46	322	56+69+…+322	NIST	
n-C25:1	C25H50	350	56+69+…+350	NIST	
n-C29:1	C29H58	406	56+69+…+406	NIST	
n-C31:1	C31H62	434	56+69+…+434	NIST	
n-C33:1	C33H66	462	56+69+…+462	NIST	
n-C22:1	C22H44	308	56+69+…+308	NIST	C22 to C26 (even n°)
n-C24:1	C24H48	336	56+69+…+336	NIST	
n-C26:1	C26H52	364	56+69+…+364	NIST	
***n*-alkanes**					
n-C14:0	C14H30	198	57+71+85+…+198	NIST	C14 to C20 (even n°)
n-C18:0	C18H38	254	57+71+85+…+254	NIST	
n-C20:0	C20H42	282	57+71+85+…+282	NIST	
n-C15:0	C15H32	212	57+71+85+…+212	NIST	C15 to C19 (odd n°)
n-C17:0	C17H36	240	57+71+85+…+240	NIST	
n-C19:0	C19H40	268	57+71+85+…+268	NIST	
n-C21:0	C21H44	296	57+71+85+…+296	NIST	C21 to C37 (odd n°)
n-C23:0	C23H48	324	57+71+85+…+324	NIST	
n-C25:0	C25H52	352	57+71+85+…+352	NIST	
n-C27:0	C27H56	380	57+71+85+…+380	NIST	
n-C29:0	C29H60	408	57+71+85+…+408	NIST	
n-C31:0	C31H64	436	57+71+85+…+436	NIST	
n-C37:0	C37H76	521	57+71+85+…+521	NIST	
n-C22:0	C22H46	311	57+71+85+…+310	NIST	C22 to C38 (even n°)
n-C24:0	C24H50	338	57+71+85+…+338	NIST	
n-C32:0	C32H66	450	57+71+85+…+450	NIST	
n-C34:0	C34H70	480	57+71+85+…+480	NIST	
n-C36:0	C36H74	506	57+71+85+…+506	NIST	
n-C38:0	C38H78	535	57+71+85+…+535	NIST	
**Alkanones**					
2-Pentadecanone	C15H30O	226	43+**58**+71	NIST	Alkanones
Heptadecanone	C17H34O	254	43+**58**+71+254	NIST	
Alkanone	Unknown	x	43+**58**+71	NIST	
2-Pentanone	C5H10O	86	**43**+86	NIST	
**Other aliphatics**					
2-Pentene, 2,4-dimethyl-	C7H14	98	41+**55**+83+98	NIST	Other aliphatics
Nonanal	C9H18O	142	29+43+43+56+**57**+70+98	NIST	
**Phenols**					
Phenol	C6H6O	94	39+65+66+**94**	NIST	Phenols
Phenol, 4-methyl-	C7H8O	108	77+79+107+108	NIST	
Phenol, -dimethyl-	C7H8O	122	77+91+**107**+121+ **122**	NIST	
**S compounds**					
2(3H)-Benzothiazolone	C7H5NOS	151	96+123+**151**	NIST	S compounds
Benzenesulfonamide, N-butyl-	C10H15NO2 S	213	51+**77**+141+170	NIST	
**Steroids**					
Stigmasta-3,5-dien-7-one	C29H46O	411	161+**174**+187+395+**410**	NIST	Steroids

Name	Formula	M	Specific mass fragments	Ref	Compound sub-groups
**(Poly)aromatics**					
Toluene	C7H8	92	**91**+92	NIST	Toluene
Benzoic acid	C7H6O2	122	51+77+**105**+122	NIST	Benzoic acid
Naphthalene, 2-phenyl-	C16H12	204	101+**204**	NIST	Polyaromatics
Phenanthrene, 3,6-dimethyl-	C16H14	206	102+191+**206**	NIST	
Retene or Phenanthrene, 3,4,5,6-tetramethyl-	Both C18H18	234	165+178+**204+219+234**	NIST	
1,4-Diphenyl-1,3-butadiene	C16H14	206	91+128+191+**206**	NIST	(Alkyl)benzene s
Alkyl-Benzene C12	C18H30	246	**91+92**+…246	NIST	
Alkyl-Benzene C16	C22H38	302	**91+92**+…302	NIST	
Alkyl-Benzene C17	C23H40	316	**91+92**+…316	NIST	
Alkyl-Benzene C18	C24H42	330	**91+92**+…330	NIST	
Alkyl-Benzene C22	C28H50	386	**91+92**+…386	NIST	

[1] Chen et al., (2009) *Journal of Food Science*, 74: 100-105; [2] Fabbri et al., (2012) *Journal of Analytical and Applied Pyrolysis*, 95: 145-155

Supplementary Method 2: Sulfur isotope analysis

All precipitation samples used for the analysis of sulfur (S) isotopes were prepared in a Class 10 clean laboratory. Concentrated nitric acid (HNO_3, Primar Plus^{TM}, Thermo Scientific) and hydrochloric acid (HCl, Primar Plus^{TM}, Thermo Scientific) used for sample preparation were further purified by a two-step sub-boiling distillation (DST 4000, Savillex). All plastic labware was pre-cleaned with HNO_3 and then rinsed with ultrapure water (≥18.2 MΩcm, Merck Millipore).

S isotopes were analysed following the method by Fassbender et al.[33], including offline S isolation by anion exchange chromatography and analysis by multicollector ICP-MS (MC-ICP-MS, Neptune Plus Thermo Scientific). Briefly, we digested an Ag_2S International Atomic Energy Agency standards (IAEA-S-1, S-2, S-3) and the precipitation samples following Craddock et al.[34]. This procedure included the addition of 5 mL of concentrated HNO_3 and evaporation to dryness at 70°C, followed by addition of 3 mL of concentrated HNO_3 and 2 mL of HCL (6 $mol \cdot L^{-1}$) and again evaporation to dryness at 70°C. The dry residue was re-dissolved in 1 mL of 0.03 $mol \cdot L^{-1}$ HNO_3 and purified using an anion exchange resin (AG1X8; Cl form; 200–400 mesh; BIORAD labs) following the protocol by Das et al.[35]. Recovery was 100 ± 2.6% (n=4)for samples containing 4 µg of S. The concentrations of two procedural blanks were 14 and 20 ng S, corresponding to 0.5% of the recovered 4 µg S.

All solutions for MC-ICP-MS analysis were diluted to a final S concentration of ~400 $\mu g \cdot L^{-1}$ in 0.3 $mol \cdot L^{-1}$ HNO_3 with the addition of 0.9 $mg \cdot L^{-1}$ of Na (prepared from 1000 $mg \cdot L^{-1}$ $NaNO_3$, TraceCERT®, Merck) to improve transmission of S thought the membrane desolvation system. All instrumental parameters for the S analysis are given in Supplementary Table 9. Background correction and delta calculation was performed with the Neptune software. Instrumental conditions were optimized using the IAEA S-2 and S-3 standards. A certified S standard (ICP-S, prepared from 1000 $mg \cdot L^{-1}$ H_2SO_4, Certipur®, Merck) was used as a bracketing standard for all samples to correct for instrument drift and mass bias. At medium resolution mode 400 $\mu g \cdot L^{-1}$ of S generated around 10 V (for ^{32}S) intensity. The intensity of the isotopes was measured on the lower mass shoulder.

Supplementary Table 9. MC-ICP-MS operating conditions for S isotope analysis.

**MC-ICP-MS**	**Neptune Plus (Thermo Scientific)**
RF power	1200 W
Cones	Ni sampler and Ni skimmer cone
Cooling gas	16 $L \cdot min^{-1}$
Auxiliary gas	0.8 $L \cdot min^{-1}$
Sample gas	0.9 $L \cdot min^{-1}$
Nebulizer	Microflow PFA-ST (100 $\mu L \cdot min^{-1}$)
Cup configuration	L3 (^{32}S), C (^{33}S), H3 (^{34}S)
Resolution mode	Medium
Measurement settings	40 cycles with 4.194 $s \cdot cycle^{-1}$
**Desolvation system**	**Apex-Ω (Elemental Scientific)**
Sweep gas	3.8 $L \cdot min^{-1}$
Nitrogen gas	6 $mL \cdot min^{-1}$
Spray chamber	Quartz, 140°C
Peltier cooling	Quartz, 3°C
Membrane	155°C

Two normalizations were applied for the conversion to the Vienna Canyon Diablo Troilite (VCDT) scale: conversion from working standard S-ICP to the certified reference material IAEA-S-1, then to the VCDT scale. The scale is defined by assigning a $\delta^{34}S$ value of =-0.3‰ to the IAEA-S-1 relative to VCDT ($\delta^{34}S_{VCDT}^{IAEA\,S-1}$), without uncertainty[36]. $\delta^{34}S$ values of samples relative to the VCDT scale ($\delta^{34}S_{VCDT}^{smp}$) are calculated using the following equation (1):

$$\delta^{34}S_{VCDT}^{smp}\ (‰) = \left[\left(\frac{\delta^{34}S_{S-ICP}^{smp}+1}{\delta^{34}S_{S-ICP}^{IAEA\,S-1}+1}\right)\times\left(\delta^{34}S_{VCDT}^{IAEA\,S-1}+1\right)-1\right]\times 1000 \qquad (1)$$

The combined uncertainty (2σ) corresponding to repeatability (n = 11) and to these two normalizations was 0.24‰.

Supplementary References

1. Breuninger ES*, et al.* Influences of sources and weather dynamics on atmospheric deposition of Se species and other trace elements. *Atmos Chem Phys* **24**, 2491-2510 (2024).

2. Huang J-H, Matzner E. Biogeochemistry of Organic and Inorganic Arsenic Species in a Forested Catchment in Germany. *Environmental Science & Technology* **41**, 1564-1569 (2007).

3. Savage L*, et al.* Elevated Trimethylarsine Oxide and Inorganic Arsenic in Northern Hemisphere Summer Monsoonal Wet Deposition. *Environmental Science & Technology* **51**, 12210-12218 (2017).

4. Savage L, Carey M, Williams PN, Meharg AA. Maritime Deposition of Organic and Inorganic Arsenic. *Environmental Science & Technology* **53**, 7288-7295 (2019).

5. Tziaras T, Pergantis SA, Stephanou EG. Investigating the Occurrence and Environmental Significance of Methylated Arsenic Species in Atmospheric Particles by Overcoming Analytical Method Limitations. *Environmental Science & Technology* **49**, 11640-11648 (2015).

6. Tanda S, Gingl K, Ličbinský R, Hegrová J, Goessler W. Occurrence, Seasonal Variation, and Size Resolved Distribution of Arsenic Species in Atmospheric Particulate Matter in an Urban Area in Southeastern Austria. *Environmental Science & Technology* **54**, 5532-5539 (2020).

7. Tanda S, Ličbinský R, Hegrová J, Faimon J, Goessler W. Arsenic speciation in aerosols of a respiratory therapeutic cave: A first approach to study arsenicals in ultrafine particles. *Science of The Total Environment* **651**, 1839-1848 (2019).

8. Jakob R*, et al.* Atmospheric stability of arsines and the determination of their oxidative products in atmospheric aerosols (PM10): evidence of the widespread phenomena of biovolatilization of arsenic. *Journal of Environmental Monitoring* **12**, 409-416 (2010).

9. Sadee BA, Foulkes ME, Hill SJ. An evaluation of extraction techniques for arsenic in staple diets (fish and rice) utilising both classical and enzymatic extraction methods. *Food Additives & Contaminants: Part A* **33**, 433-441 (2016).

10. Sun G-X*, et al.* Survey of arsenic and its speciation in rice products such as breakfast cereals, rice crackers and Japanese rice condiments. *Environment International* **35**, 473-475 (2009).

11. Xie J*, et al.* Distribution and chemical speciation of arsenic in different sized atmospheric particulate matters. *Journal of Environmental Sciences* **108**, 1-7 (2021).

12. González-Castanedo Y*, et al.* Arsenic species in atmospheric particulate matter as tracer of the air quality of Doñana Natural Park (SW Spain). *Chemosphere* **119**, 1296-1303 (2015).

13. Raber G, Stock N, Hanel P, Murko M, Navratilova J, Francesconi KA. An improved HPLC–ICPMS method for determining inorganic arsenic in food: Application to rice, wheat and tuna fish. *Food Chemistry* **134**, 524-532 (2012).

14. Larsen EH, Stürup S. Carbon-enhanced inductively coupled plasma mass spectrometric detection of arsenic and selenium and its application to arsenic speciation. *Journal of Analytical Atomic Spectrometry* **9**, 1099-1105 (1994).

15. Guo X, Sturgeon RE, Mester Z, Gardner GJ. Photochemical alkylation of inorganic arsenic Part 1. Identification of volatile arsenic species. *Journal of Analytical Atomic Spectrometry* **20**, 702-708 (2005).

16. Madronich S, McKenzie RL, Björn LO, Caldwell MM. Changes in biologically active ultraviolet radiation reaching the Earth's surface. *Journal of Photochemistry and Photobiology B: Biology* **46**, 5-19 (1998).

17. Kieber RJ, Peake B, Willey JD, Avery GB. Dissolved organic carbon and organic acids in coastal New Zealand rainwater. *Atmospheric Environment* **36**, 3557-3563 (2002).

18. Sun X*, et al.* Organic acids in cloud water and rainwater at a mountain site in acid rain areas of South China. *Environmental Science and Pollution Research* **23**, 9529-9539 (2016).

19. Peña RM, García S, Herrero C, Losada M, Vázquez A, Lucas T. Organic acids and aldehydes in rainwater in a northwest region of Spain. *Atmospheric Environment* **36**, 5277-5288 (2002).

20. Gioda A*, et al.* Speciation of water-soluble inorganic, organic, and total nitrogen in a background marine environment: Cloud water, rainwater, and aerosol particles. *Journal of Geophysical Research: Atmospheres* **116**, (2011).

21. Löflund M, Kasper-Giebl A, Schuster B, Giebl H, Hitzenberger R, Puxbaum H. Formic, acetic, oxalic, malonic and succinic acid concentrations and their contribution to organic carbon in cloud water. *Atmospheric Environment* **36**, 1553-1558 (2002).

22. Sugo T*, et al.* The role of humic-like substances as atmospheric surfactants in the formation of summer-heavy rainfall in downtown Tokyo. *City and Environment Interactions* **3**, 100022 (2019).

23. Iakovleva VP. UV spectrophotometric studies of arsenic (III) and antimony (III) aqueous chemistry from 25 to 300 C.). ETH Zurich (2003).

24. Ruderman G, Caffarena ER, Mogilner IG, Tolosa EJ. Hydrogen Bonding of Carboxylic Acids in Aqueous Solutions—UV Spectroscopy, Viscosity, and Molecular Simulation of Acetic Acid. *Journal of Solution Chemistry* **27**, 935-948 (1998).

25. Sugarman B. The ultra-violet absorption spectrum of formic acid. *Proceedings of the Physical Society* **55**, 429 (1943).

26. Lund Myhre CE, Nielsen CJ. Optical properties in the UV and visible spectral region of organic acids relevant to tropospheric aerosols. *Atmos Chem Phys* **4**, 1759-1769 (2004).

27. Chen J, Rosen BP. Arsenite Methyltransferase Diversity and Optimization of Methylation Efficiency. *Environmental Science & Technology* **57**, 9754-9761 (2023).

28. Zhao F-J*, et al.* Arsenic Methylation in Soils and Its Relationship with Microbial arsM Abundance and Diversity, and As Speciation in Rice. *Environmental Science & Technology* **47**, 7147-7154 (2013).

29. Lomax C*, et al.* Methylated arsenic species in plants originate from soil microorganisms. *New Phytologist* **193**, 665-672 (2012).

30. Reid MC, Maillard J, Bagnoud A, Falquet L, Le Vo P, Bernier-Latmani R. Arsenic Methylation Dynamics in a Rice Paddy Soil Anaerobic Enrichment Culture. *Environmental Science & Technology* **51**, 10546-10554 (2017).

31. Viacava K*, et al.* Variability in Arsenic Methylation Efficiency across Aerobic and Anaerobic Microorganisms. *Environmental Science & Technology* **54**, 14343-14351 (2020).

32. Tolu J, Gerber L, Boily J-F, Bindler R. High-throughput characterization of sediment organic matter by pyrolysis–gas chromatography/mass spectrometry and multivariate curve resolution: A promising analytical tool in (paleo)limnology. *Analytica Chimica Acta* **880**, 93-102 (2015).

33. Faßbender S*, et al.* Species-specific isotope dilution analysis of monomethylmercury in sediment using GC/ICP-ToF-MS and comparison with ICP-Q-MS and ICP-SF-MS. *Analytical and bioanalytical chemistry* **413**, 5279-5289 (2021).

34. Craddock PR, Rouxel OJ, Ball LA, Bach W. Sulfur isotope measurement of sulfate and sulfide by high-resolution MC-ICP-MS. *Chemical Geology* **253**, 102-113 (2008).

35. Das A, Chung C-H, You C-F, Shen M-L. Application of an improved ion exchange technique for the measurement of δ34S values from microgram quantities of sulfur by MC-ICPMS. *Journal of Analytical Atomic Spectrometry* **27**, 2088-2093 (2012).

36. Coplen TB, Krouse HR. Sulphur isotope data consistency improved. *Nature* **392**, 32-32 (1998).
