# Supplementary material for: Marine and terrestrial contributions to atmospheric deposition fluxes of methylated arsenic species
Source: Nat Commun. 2024 Nov 7;15:9623. doi: 10.1038/s41467-024-53974-z (PMC11543862; doi:10.1038/s41467-024-53974-z)
Supplement: Supplementary file 2 — Description of Additional Supplementary Files [file 41467_2024_53974_MOESM2_ESM.pdf]

Description of Additional Supplementary Files:

Supplementary Dataset 1: Generated data on arsenic speciation, sulfur isotopes, dissolved organic carbon, as well as modelled moisture sources and time spend in the boundary layer.
